# The transcription factor Stat-1 is essential for Schwann cell differentiation, myelination and myelin sheath regeneration

**DOI:** 10.1186/s10020-023-00667-w

**Published:** 2023-06-26

**Authors:** Jinghui Xu, Bin Zhang, Jieyi Cai, Qianqian Peng, Junxia Hu, Parizat Askar, Jianghong Shangguan, Wenfeng Su, Changlai Zhu, Hualin Sun, Songlin Zhou, Gang Chen, Xiaoming Yang, Yun Gu

**Affiliations:** Jiangsu Key Laboratory of Neuroregeneration, Co-Innovation Center of Neuroregeneration, Jiangsu Clinical Medicine Center of Tissue Engineering and Nerve Injury Repair, Nantong University, Nantong, Jiangsu 226001 People’s Republic of China

**Keywords:** Stat1, Schwann cells, Differentiation, Myelination and remyelination, Demyelinating diseases

## Abstract

**Background:**

Myelin sheath is a crucial accessory to the functional nerve-fiber unit, its disruption or loss can lead to axonal degeneration and subsequent neurodegenerative diseases (NDs). Notwithstanding of substantial progress in possible molecular mechanisms underlying myelination, there is no therapeutics that prevent demyelination in NDs. Therefore, it is crucial to seek for potential intervention targets. Here, we focused on the transcriptional factor, signal transducer and activator of transcription 1 (Stat1), to explore its effects on myelination and its potential as a drug target.

**Methods:**

By analyzing the transcriptome data obtained from Schwann cells (SCs) at different stages of myelination, it was found that Stat1 might be involved in myelination. To test this, we used the following experiments: (1) In vivo, the effect of Stat1 on remyelination was observed in an in vivo myelination mode with Stat1 knockdown in sciatic nerves or specific knockdown in SCs. (2) In vitro, the RNA interference combined with cell proliferation assay, scratch assay, SC aggregate sphere migration assay, and a SC differentiation model, were used to assess the effects of Stat1 on SC proliferation, migration and differentiation. Chromatin immunoprecipitation sequencing (ChIP-Seq), RNA-Seq, ChIP-qPCR and luciferase activity reporter assay were performed to investigate the possible mechanisms of Stat1 regulating myelination.

**Results:**

Stat1 is important for myelination. Stat1 knockdown in nerve or in SCs reduces the axonal remyelination in the injured sciatic nerve of rats. Deletion of Stat1 in SCs blocks SC differentiation thereby inhibiting the myelination program. Stat1 interacts with the promoter of Rab11-family interacting protein 1 (Rab11fip1) to initiate SC differentiation.

**Conclusion:**

Our findings demonstrate that Stat1 regulates SC differentiation to control myelinogenic programs and repair, uncover a novel function of Stat1, providing a candidate molecule for clinical intervention in demyelinating diseases.

**Supplementary Information:**

The online version contains supplementary material available at 10.1186/s10020-023-00667-w.

## Introduction

Myelin sheath is essential for maintaining nervous system function, and is formed by Schwann cells (SCs) in the peripheral nervous system (PNS). It consists of multiple layers of specialized membranes that wrap axons to facilitate the rapid propagation of action potentials and give axons proper nutrition (Salzer and Zalc 2016). Failure of SCs to myelinate or remyelinate axons disrupts the insulating conduction of nerve electrical signals, resulting in motor and sensory function deficits and even axonal degeneration, and hence to severe neurological disability and neurodegenerative diseases such as Charcot–Marie–Tooth disease (Saab and Nave 2017; Monje 2018). To date, there are no therapeutic interventions that directly prevent demyelination in myelinopathies and related diseases, especially as the precise mechanisms underlying myelin degeneration and regeneration remain undetermined (Pareyson et al. 2017; Miniou and Fontes 2021). SCs, as a crucial role in myelin sheath in PNS, their specification, lineage progression, differentiation, myelin formation and maintenance are of most importance to prevent demyelination, preserve axonal integrity and restore neuronal function (Zhou and Notterpek 2016; Villoslada and Martinez-Lapiscina 2019). Therefore, seeking for the regulate factors controlling the process of SC differentiation and myelin formation is of great significance for the treatment of neurological diseases.

SC lineage progression, axon sorting, differentiation and myelination are coordinated by a number of transcriptional factors (TFs), including positive regulators that promote SC myelination such as Brn2, Sox10, Pou3f1 (also known as Oct6), Yy1 and Krox20 (also known as Egr2) and negative regulators that inhibit myelination such as Sox2, Notch, Zeb2 and Id2 (Mirsky and Jessen 1996; Monk et al. 2015; Quintes et al. 2016; Roberts et al. 2017; Torii et al. 2019). The main axis of these TFs is the activation of Oct6 by Sox10, which then cooperates with Oct6 to induce Egr2 expression, thereby enabling the sequential progression of SC development in the order of immature to promyelinating SCs, and finally into myelinating SCs (Svaren and Meijer 2008; Stolt and Wegner 2016; Sock and Wegner 2019). Despite substantial progress in identifying the underlying TFs that regulate myelination, considering that the known TFs and their interactions may represent only a small proportion of regulatory mechanisms that induce and inhibit SC development and myelination, therefore challenges remain and more work is needed to find other key TFs.

Recently, multiple groups have performed high-throughput screens to identify genes and associated pathways that regulate axon regeneration and myelination (Nagarajan et al. 2002; Balakrishnan et al. 2016; Kangas et al. 2016). Our laboratory has conducted transcriptome analysis of SCs at various stages of myelination (i.e. immature, promyelinating and myelinating SCs) in an in vitro myelination model [aka SCs and dorsal root ganglion (DRG) neurons coculture myelination model], and identified 17 potential TFs regulating myelination including known transcriptional activator such as Nab2 and Egr2, etc., unknown TFs such as signal transducer and activator of transcription 1 (Stat1) and Sin3A etc.(Zhang et al. 2022). Stat1, a member of a Stats proteins family, has been extensively reported to be important in the regulator of infection, inflammation, and T-cell differentiation (Levy and Darnell 2002; Shuai 2003; Kang et al. 2019; Huffaker et al. 2021). Recent years, some studies have reported that Stat1 regulate neuroimmune responses in the central nervous system (CNS) by triggering the activation of Janus kinase (JAK)/Stat1 in microglia and astrocytes by binding of interferon gamma (IFN γ) to its receptors (Przanowski et al. 2014; Hidano et al. 2016; Gaojian et al. 2020). About Stat1 function in myelination, there are only few reports. Lin et al. demonstrated that enforced expression of IFN-g in demyelinating lesions of the CNS inhibited remyelination in this areas in a Stat1-dependent and Stat1-independent manner, and found that Stat1 deletion resulted in oligodendrocyte loss leading to a decrease of myelinated axons (Lin and Lin 2010). Notably, little is known about whether Stat1 affects myelination and how it functions in myelination and remyelination.

Here, we focused on the role of Stat1 in the myelination of SCs in the PNS and found that it plays a critical role in the lineage progression of SCs during myelination and remyelination after nerve injury. Stat1 depletion in SCs dramatically impairs the SC differentiation and myelin production after demyelination injury. Using RNA interference combined with chromatin immunoprecipitation sequencing (ChIP-Seq) and RNA sequencing (RNA-Seq), we found that Stat1 affects the transcriptional landscape of SCs during differentiation. Furthermore, we revealed that Stat1 initiates or regulates SC differentiation and myelination, at least in part, by binding to the promoter of its target gene Rab11fip1. Our findings uncover a novel function of Stat1 to regulate SC differentiation and myelination, providing a candidate molecular target for clinical interventions such as drug design in demyelinating diseases.

## Materials and methods

### Transcriptomic data (GSE163132) analysis

Weighted gene co-expression network analysis (WGCNA) were performed to reanalyzed our previous transcriptomic data (GEO Series accession number GSE163132) by using the ‘cutreeHybrid’ function from the R package as previously (Zhang et al. 2022). The resulting 8 time-specific modules (size > 50 genes, corresponding correlation with time point > 0.6 and a P-value ≤ 0.005) were subjected to identify the biological function using the Gene Ontology (GO) enrichment analyses (Additional file 2: Table S1.1–1.3). We then screened for the transcription factors (TFs) in each module that had an expression fold change ≥ 1.5 and were involved in regulating myelination. The list of TFs are provided in Additional file 2: Table S1.4.

### Animals, nerve injury and virus injection

Newborn and adult Sprague–Dawley (SD) rats were obtained from the Experimental Animal Center of Nantong University. All animal use and studies were conducted in accordance with relevant ethical regulations and were approved by the Nantong University Administration Committee of Experimental Animals.

For virus injection and nerve injury, adult rats at 8 weeks were under deep anesthesia with 3% isofluorane. The sciatic nerve of rat left leg was exposed, and the adeno-associated virus (AAV2/8; Obio Technology, Shanghai, China) carrying Stat1-shRNA (final titer of 5 × 10^8^, 4 μl) or scrambled sequence (final titer of 5 × 10^8^, 4 μl) were injected into sciatic nerve with a micro-syringe as described before (Gonzalez et al. 2014; Chen et al. 2019), and the needle was slowly withdrawn after keeping still for 2 min to ensure that the virus was remained in the nerve tissue. Sciatic nerve crush experiments were performed 3 weeks post virus injection. Expose the sciatic nerve as described above and crush for 30 s with a fine hemostat. In sham-operated rats, the sciatic nerve was exposed without damaging the nerve tissue. We maintained the rats' body temperature with a heating pad throughout the surgery and during recovery from anesthesia. After fully awake, animals were kept in cages (3 per cage) according to groups, housed and fed routinely, and their status was monitored regularly. Neural tissue were collected 21 days after virus injection or 21 days after sciatic nerve crush for immunohistochemistry (IHC), western blotting (WB), or transmission electron microscopy (TEM).

### Culture and purification of SCs

SCs were cultured and purified as previously described (Gu et al. 2014). Sciatic nerves were harvested from neonatal rats (1–2 days), cut into small pieces and enzymatically treated with 1% collagenase (17018-029, Gibco, Carlsbad, CA, USA) and 0.125% trypsin (25200-056, Gibco). The cell mixture was then resuspended in DMEM (11965-092, Gibco), 10% FBS (12483-020, Gibco), and penicillin–streptomycin (PS; 15140122, Thermo Fisher Scientific, Cleveland, OH, USA) using a 400-mesh cell screen to make a single-cell suspension, seeded in 50 μg/ml poly-d-lysine (PDL; P-7890, Sigma, St Louis, MO, USA) coated dishes with 10 mM cytosine arabinoside (C6645, Sigma) and 10% FBS in DMEM for 1 day to remove fibroblasts. Afterwards, the medium was changed to supplemented with 5 μM forskolin (F6886, Sigma), 2 ng/ml Neuregulin 1 (Nrg1; 396-HB-050, R&D, MN, USA) and 10% FBS in DMEM. After 48 h, cells were incubated with anti-Thy1 antibody (1:1000, M7898, Sigma) to remove remaining contaminating fibroblasts. SC purity was assessed by S100β (1:500, S2532, Sigma) immunostaining. Passage 2 SCs with > 95% purity were used for all cell experiments.

### Culture and purification of DRG explants

DRG from embryonic day (E) 15 rat were plated as explants on PDL-coated coverslips. After adhering within 24 h in the DMEM-HG medium (11965-084, Gibco) containing 10% FBS, cultures were maintained in serum-free neurobasal medium (NB; 21103-049, Gibco), 50 ng/ml NGF (N2513, Sigma), 2% B27 supplement (A35828-01, Gibco), and 2 mM l-glutamine (J60573.14, Gibco). After 48 h of culture, the medium was changed to DRG neuron purification medium consisting of NB medium, uridine (U6381, Sigma) and 5-fluorodeoxyuridine (F0503, Sigma) to remove non-neuronal cells. After being cycled on neuron purification medium for three times, > 95% DRG neuron purity was achieved, assessed by β-III tubulin (TuJ1, 1:500, ab18207, Abcam, Cambridge, England) staining.

### SC-DRG myelinating coculture

Approximately 50,000 SCs were seeded in the purified DRG neurons, and cocultured with DMEM-HG medium containing 10% FBS, 0.4% glucose (15023021, Gibco), 2 mM l-glutamine and 50 ng/ml NGF. After 3 days of co-cultivation, basal layer formation was initiated for approximately 4 days with DMEM medium supplemented with 0.2% bovine serum albumin (BSA; V900933, Sigma), ITS (I3146, Sigma) and 50 ng/ml NGF, and then the medium were changed to DMEM medium containing 50 μg/ml l-ascorbic acid (A0278, Sigma), 50 ng/ml NGF and 15% FBS to induce myelination for 2–3 weeks until myelin sheath being achieved, with fresh media provided every 2 days. The myelin associated glycoprotein (MAG) immunostaining was performed to assess myelination in the co-culture system.

### SC differentiation assay

SC differentiation was induced as previously described (Gu et al. 2018). In brief, about 10,000 SCs were seeded into PDL-coated 24-well culture plates and cultured in DMEM containing 10% FBS for 24 h. The medium was then changed to DMEM/F12 (11320-033, Gibco) supplement with 1% FBS, 20 ng/ml Nrg1, and 1 mM dibutyryl cyclic AMP (db-cAMP; D0627, Sigma) to initiate differentiation. After 3 days of induction, WB and immunocytochemistry (ICC) were used to analyze the expression of MAG and myelin protein zero (P0 or MPZ) in myelinated/unmyelinated SCs to determine whether the cells had acquired a differentiated phenotype.

### SC migration assays

Two methods were used to detect cell migration: (1) cell scratch assay and (2) cell spheroid migration assay. For the former, 5,000 SCs were seeded into culture insert (80242, Ibidi, DE, Germany) on a 24-well plate and cultured with DMEM supplement with 10% FBS for 24 h. After removing the culture insert, two separate islands of cells were obtained. The medium was then changed to DMEM containing 2 μg/ml mitomycin C (M5353, Sigma) and 10% FBS. Following 12 h of incubation, cell images were collected by microscope (Leica Microsystems, Bensheim, Germany) and cell migration distance was measured using NIH Image J 1.46R. For the latter, SC spheres were seeded onto dorsal root ganglion axons to observe their migration as described previously (Yamauchi et al. 2004). Briefly, 5000 SCs were cultured in suspension, shake gently every 2 h to prevent them adherence the dishes and obtain SC spheres after 24 h. The cell spheroids were then seeded on the fascicular axons of DRG neurons for 12 h and then photographed under a microscope, and the migration distance of the cells out of the spheroids was measured using analysis software Image J.

### SC proliferation assay

We used the Cell-Light EdU Apollo643 kit (C10310, Ribobio, Guangzhou, China) to detect the proliferation of SCs cultured on two substrates (i.e. fascicular DRG neurites and PDL). Briefly, approximately 40,000 cells were seeded onto fascicular axons or PDL-coated coverslips for 24 h, followed by 50 µM EdU treatment for an additional 24 h. Cells were then fixed with 4% formaldehyde for 0.5 h and EdU staining was performed according to the instructions of manufacturer, and nuclei were labeled with Hoechst 33342 (14533, 1 µg/ml, Sigma) to determine the total cell number. The percentage of cell proliferation was calculated after 10 randomly selected regions per well were captured using a fluorescence microscope (Leica), and the experiment was repeated at least 3 times.

### Short interfering RNA transfection and lentivirus infection

For short interfering RNA (siRNA) transfection, approximately 1 × 10^5^ cells SCs were seeded in 24-well culture plates and cultivated for 24 h. The siRNAs including Stat1-siRNAs or Rab11fip1-siRNAs, and their non-targeting negative control (RiboBio) were transfected into cells using Lipofectamine RNAiMAX Transfection Reagent (Invitrogen, Carlsbad, CA, USA). All siRNAs were used at a 10 nM concentration and their sequences are provided in Additional file 5: Table S4.1. Forty eight hours post-transfection, SCs were used for subsequent experiments such as WB to test knockdown efficiency, cell proliferation, migration and differentiation.

For lentivirus (LV) infection, approximately 1 × 10^5^ cells SCs were seeded in 24-well culture plates and cultivated for 24 h. LVs carrying Stat1-shRNA or a non-targeting negative control were added to the cell culture at a multiplicity of infection (MOI) of 20 with 6 μg/ml of polybrene. After 12 h of incubation, the supernatant was discarded and fresh DMEM medium and 10% FBS were added, and cultured for 72 h. The obtained Stat1 knockdown SCs were used for subsequent experiments, such as protein extraction and WB verification of transfection efficiency, or co-culture with neurons to form myelin sheaths.

### RNA isolation and RT-qPCR

Total RNA was extracted from cells or sciatic nerves using the RNeasy Plus Mini Kit (74136, Qiagen, CA, USA), complementary DNA was obtained by reverse transcription using the Superscript Kit (11904018, Invitrogen CA, USA), and then SYBR green PCR mix (A46012, Life Technologies, Gaithersburg, MD, USA) was used to perform quantitative real-time PCR (qRT-PCR). Data analysis was performed using the ^ΔΔ^CT method. Primer sequences can be found in Additional file 5: Table S4.2.

### Western blotting analysis

Cells or minced sciatic nerves were mixed with RIPA lysis buffer (P0013B, Beyotime, Shanghai, China) at 4 °C for 10 min or 30 min, respectively. The lysate was centrifuged at 13,200 rpm at 4 °C for 15 min and the supernatant was quantified using the BCA protein quantification kit (P0012, Beyotime). The 20–30 µg of protein were used for sodium dodecyl sulfate–polyacrylamide gel electrophoresis (SDS-PAGE). After blotting onto PVDF membranes (Millipore, Bedford, MA, USA), the membranes were blocked with 5% nonfat milk for 1 h at room temperature and then incubated overnight at 4 °C with the primary antibodies as follow: Stat1 (14994, Cell Signaling, 1:1000), MAG (34-6200, Invitrogen, 1:100), Egr2 (NB110-59723, Novus, 1:500), Nab2 (PA5-75321, ThermoFisher, 1:500), P0 (NB100-1607, Novus, CO, USA; 1:500), Rab11fip1 (sc-517228, Santa Cruz, CA, USA; 1:500) and GAPDH (60004-1-Ig, Proteintech, Chicago, USA; 1:20,000). After 3 washes with TBS, the membrane was incubated with the corresponding HRP-conjugated secondary antibody for 1 h at room temperature before detection using the Pierce™ ECL Western Kit (ThermoFisher). The membranes were detected with a scanner (Bio-Rad Hercules, CA, USA) to obtain grayscale images, data analysis was processed using Image J.

### Immunocytochemistry and immunohistochemistry

Cells fixed in 4% paraformaldehyde (PFA) for 30 min at room temperature were used for immunocytochemistry (ICC). PFA-fixed tissue was embedded in optimal cutting temperature compound (OCT) and cryostat cut into tissue sections (12 µm) for IHC. The fixed cells or tissue sections were blocked in 5% donkey serum plus 0.1% Triton X-100 in PBS (PBS-T) for 1–2 h at room temperature, and then incubated overnight at 4 °C with the following primary antibodies: S100β (S2532, Sigma, 1:200), MAG (34–6200, Invitrogen, 1:100), Stat1 (14994, Cell Signaling, 1:200), beta-III tubulin (TuJ1, MMS-435P, Covance, 1:250), P0 (NB100-1607, Novus, 1:50). After 3 washes with PBS, the cells or tissue sections were incubated with corresponding fluorescence-conjugated secondary antibodies (1:1,000; Jackson Immunoresearch, PA, USA) for 1 h at room temperature. The images were captured using a fluorescence microscope (Leica).

### Transmission electron microscopy

For ultrastructural analysis, after being fixed with 2.5% glutaraldehyde and post fixed with 1% osmium tetroxide, the nerve tissues were embedded into Epon 812 epoxy resin. Tissue blocks were then ultrathinly cut into 50 nm thick sections, which were stained with lead citrate and uranyl acetate. The stained samples were observed and photographed using a transmission electron microscope (JEOL Ltd., Tokyo, Japan). For quantification, at least 50 axons were randomly selected from each nerve (n = 3 per group) for analysis of axon diameter, myelin sheath thickness and layers, as well as *G* ratio, etc.

### RNA-Seq and data analysis

RNA from si-Control and si-Stat1 SCs was used to generate sequencing libraries with the NEBNext^®^ UltraTM RNA Library Prep Kit for Illumina^®^ (New England Biolabs, MA, USA). The libraries were sequenced on the Illumina Novaseq platform and generate 150 bp paired-end reads, which were cleaned and aligned to the reference genome using Hisat2 v2.0.5, each gene reads were counted and mapped to that gene with FeatureCounts v1.5.0-p3. Based on the length of the gene, the FPKM (transcript kilobase fragments per million mapped reads) was calculated. Differential expression analysis was performed using the DESeq2 R package (1.16.1), and genes with adjusted P-values < 0.05 were designated as differential expression genes (DEGs). DEGs were subjected to GO enrichment analysis, and GO terms with P-values < 0.05 were significantly enrichment. In addition, the DEGs were further analyzed using the Gene Set Enrichment Analysis (GSEA), and the terms with |NES|> 1, NOM p-val < 0.05, and FDR q-val < 0.25 were significantly enrichment. The data have been submitted to the GEO repository (GEO Series accession number: GSE211336).

### Chromatin immunoprecipitation sequencing (ChIP-Seq)

For ChIP, 4 × 10^6^ cells (differentiated and undifferentiated SCs) were treated with fresh 1% formaldehyde (#J60401, ThermoFisher) for cell cross-linking, which was terminated with 125 mM glycine. Cells were harvested and enzymatically digested to disrupt chromatin, breaking genomic DNA to 100–500 bp. Afterwards, the chromatin fragments (~ 300 µg) were incubated with Stat1 antibody (5 μg, Cell Signaling, #14994) at 4 °C overnight, followed by the addition of ChIP Grade proteinA/G beads (20 μl, #26156, ThermoFisher) for a further 4–6 h at 4 °C. The beads were then melted with proteinase K, followed by extraction using a DNA extraction kit for DNA library construction and sequencing analysis. The prepared ChIP-Seq libraries were detected using an Illumina NovaSeq 6000, and ChIP-Seq data were read and aligned to the rat genome (Rn6) using Bowtie2 v2.3.5.1. Based on the ENCODE (v90) overlap rule, the MACS version 1.4.2 (http://liulab.dfci.harvard.edu/MACS) was used to perform peak calling with a p-value cutoff of 10–9, and used the software IGV 2.9.4 to show the peaks. Furthermore, the motifs likely to be targeted by Stat1 were predicted using the HOMER v4.11 program (http://homer.salk.edu/homer). The data have been submitted to the GEO repository (GEO Series accession number: GSE211337).

### ChIP-qPCR

Pooled differentiating Schwann cells were fixed with fresh 1% formaldehyde (J60401,ThermoFisher) for 20 min, and enzymatically lysed to break genomic DNA to 200 and 1000 bp. Sheared chromatins were incubated with 5 µg of Stat1 antibody (Cell Signaling, #14994) at 4 °C. Magna ChIP G Kit (Merck Millipore) was used to perform ChIP experiments. The DNAs obtained by Stat1 ChIP were used as the templates for quantitative real-time PCR, and the IgG group served as a control. The relative fold enrichment was calculated using the 2^−ΔCT^ method to determine the specific region bound by the Stat1 protein. Primers used to study gene promoter regions such as Ano1, Nts, C1qb and Rab11fip1 are provided in Additional file 5: Table S4.3.

### Luciferase reporter assay

Eukaryotic Promoter Database (http://epd.vitalit.ch/) was used to choose the promoter regions of four genes (Ano1, Nts, C1qb and Rab11fip1) that may bind to Stat1. The promoter region of Ano1 (chromosome 1 between positions 2,178,430,055 and 2,178,470,055, rat genome version rn6), the Nts promoter region (chromosome 7 between positions 44,118,018 and 44,123,018), the C1qb promoter region (chromosome 5 between positions 155,249,945 and 155,253,945), and the Rab11fip1 promoter region (chromosome 16 between positions 69,046,727 and 69,050,727). Wild-type and mutant DNA fragments corresponding to promoter of these genes were PCR amplified and subcloned into the pGV238 luciferase expression vector (Genechem, Shanghai, China) between the KpnI and XhoI sites. All constructs were confirmed by DNA sequencing. Primer sequences can be found in Additional file 5: Table S4.4.

HEK293 cells were used for luciferase reporter assay. Using Lipofectamine 3000 transfection reagent (L3000008, ThermoFisher), the pGV238 luciferase plasmid carried the wild-type and mutant DNA fragments corresponding to promoter of four genes, and the pGV141 plasmid containing Renilla luciferase (Genechem) loaded with the coding region of Stat1 (NM_032612) were co-transfected into HEK293 cells. After 48 h transfection, the relative luciferase activity (the ratio of firefly luciferase activity to Renilla luciferase activity) was measured using the dual luciferase assay system (Promega, Madison, WI, USA), compared to the transfected pGV238 base vector as a control.

### Statistical analysis

Prism statistical analysis software (GraphPad Prism 8.0.1, GraphPad Software Inc., CA, USA) was used to carry out statistical analysis. Analysis was performed using one-way or two-way analysis of variance (ANOVA) combined with Tukey's multiple comparison test for multi-group data, and the unpaired two-tailed Student's t-test for two-group data. P value < 0.05 is designated as significantly different and indicated as follows: *p < 0.05, **p < 0.01, and ***p < 0.001. Also unless otherwise stated, data are presented as mean ± SD.

## Results

### Screening for myelination-related TFs by analyzing the transcriptomic data of myelinating SCs

**Fig. 1:**
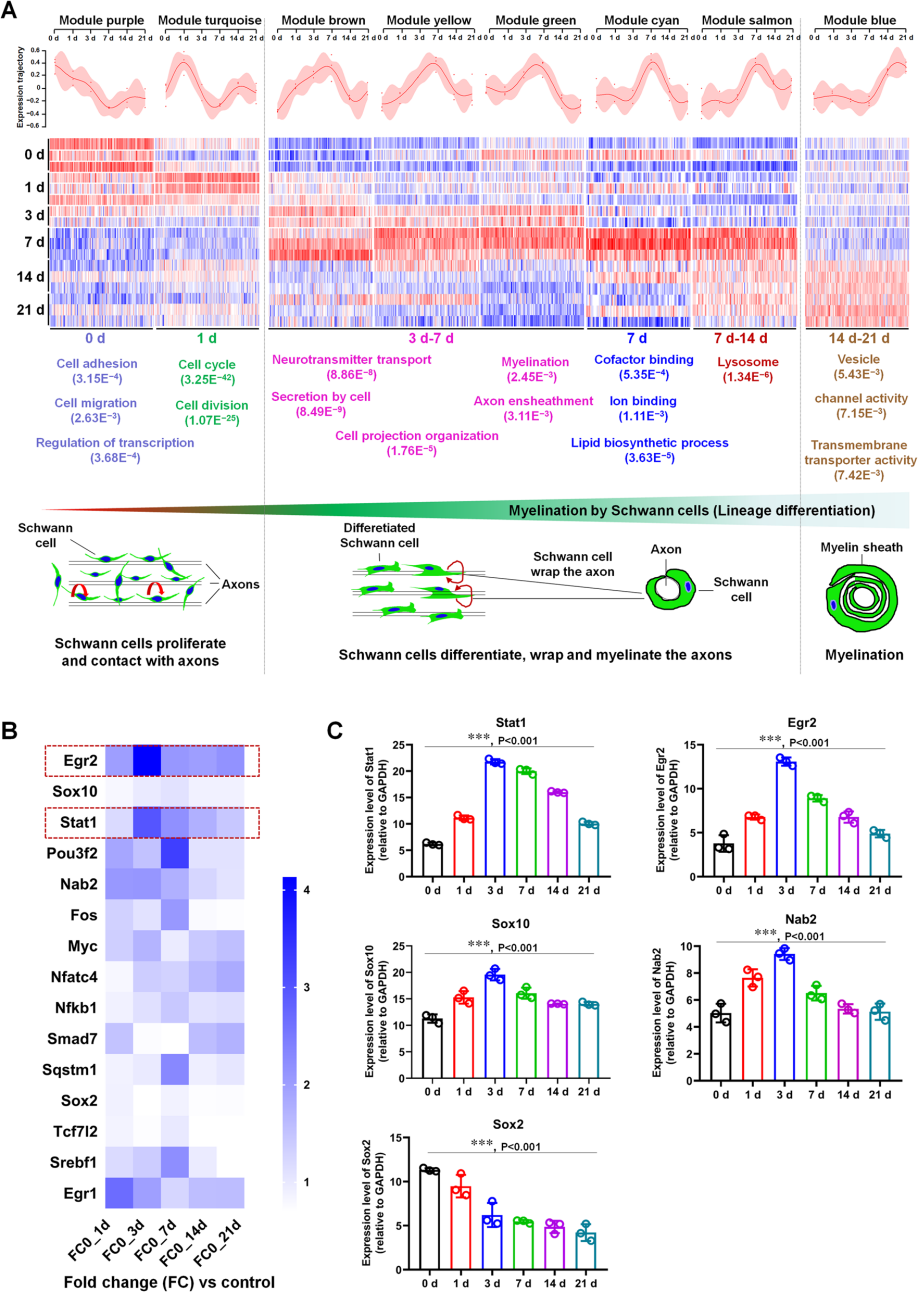
Screening for myelination-related TFs by analyzing the transcriptomic data of myelinating SCs. **A** Graphs (top) showing module eigengene expression trends. Heatmaps (middle) depicting expression of genes (columns) across samples (rows) for eight time-specific modules (purple and turquoise correspond to 0 and 1 day; green, brown, yellow, cyan s and salmon correspond to 3, 7 and 14 days; blue correspond to 14 and 21 days). The colors, from blue through white to red, indicate low to high correlations. GO analyses (bottom) showing the main biological function of the eight modules. Schematic diagram indicates the time-specific modules of SC lineage development and myelination. The gray dotted line divides it into three parts, namely SC proliferation and adhesion (0–1 day), SC differentiation, surrounding axons and myelination (3–14 days), and forming mature myelin sheath (14–21 days); red thick curved arrows indicate cell proliferation; red thin curved arrows indicate SCs wrapping axons. **B** Heat map of the changes in the expression of 15 signature TFs (rows) during SC myelination (columns). The red dotted box indicates 3 genes with the same expression trend. **C** QPCR analysis of 6 gene expression dynamic in SCs during myelination. One-way ANOVA, ***p < 0.001, n = 3 per group

Using WGCNA to analyze the transcriptomic data (GSE163132) of the SCs in the myelin co-culture system consisting of DRG neurons and SCs at six time-points (co-cultured for 0, 1, 3, 7, 14, and 21 days), the 8 time-specific modules (size > 50 genes, corresponding correlation with time point > 0.6 and *p*-value ≤ 0.005) were obtained (Additional file 2: Table S1.1–1.2). Base on the GO analyses, we found that the function of those modules correspond to the three transcriptional stages of SC myelination (Fig. 1A; Additional file 2: Table S1.3): stage I (days 0 and 1; SC proliferation; module turquoise and purple), stage II (days 3, 7, and 14; SC differentiation, promyelination and myelination; module green, brown, yellow, cyan, and salmon), and stage III (days 14 and 21; SC myelination; module blue). Next, to screen the TFs that contribute to myelination, we intersected the DEGs in stage II -specific modules with those TFs obtained from the PubMed database using the key terms "SC differentiation" and "myelination", and 14 TFs including classical regulators of myelination such as Egr2, Sox10, and Nab2 were obtained (Additional file 2: Table S1.4), in which Stat1 attracted our attention because of its possible new role in regulating myelination. Furthermore, heatmap analysis (Fig. 1B) and qRT-PCR results (Fig. 1C; Additional file 1: Fig. S1) showed that Stat1 had similar expression trends to Egr2, implicating that Stat1 may be involved in regulation of myelination.

### Knockdown of Stat1 in sciatic nerve inhibits remyelination after nerve injury

**Fig. 2:**
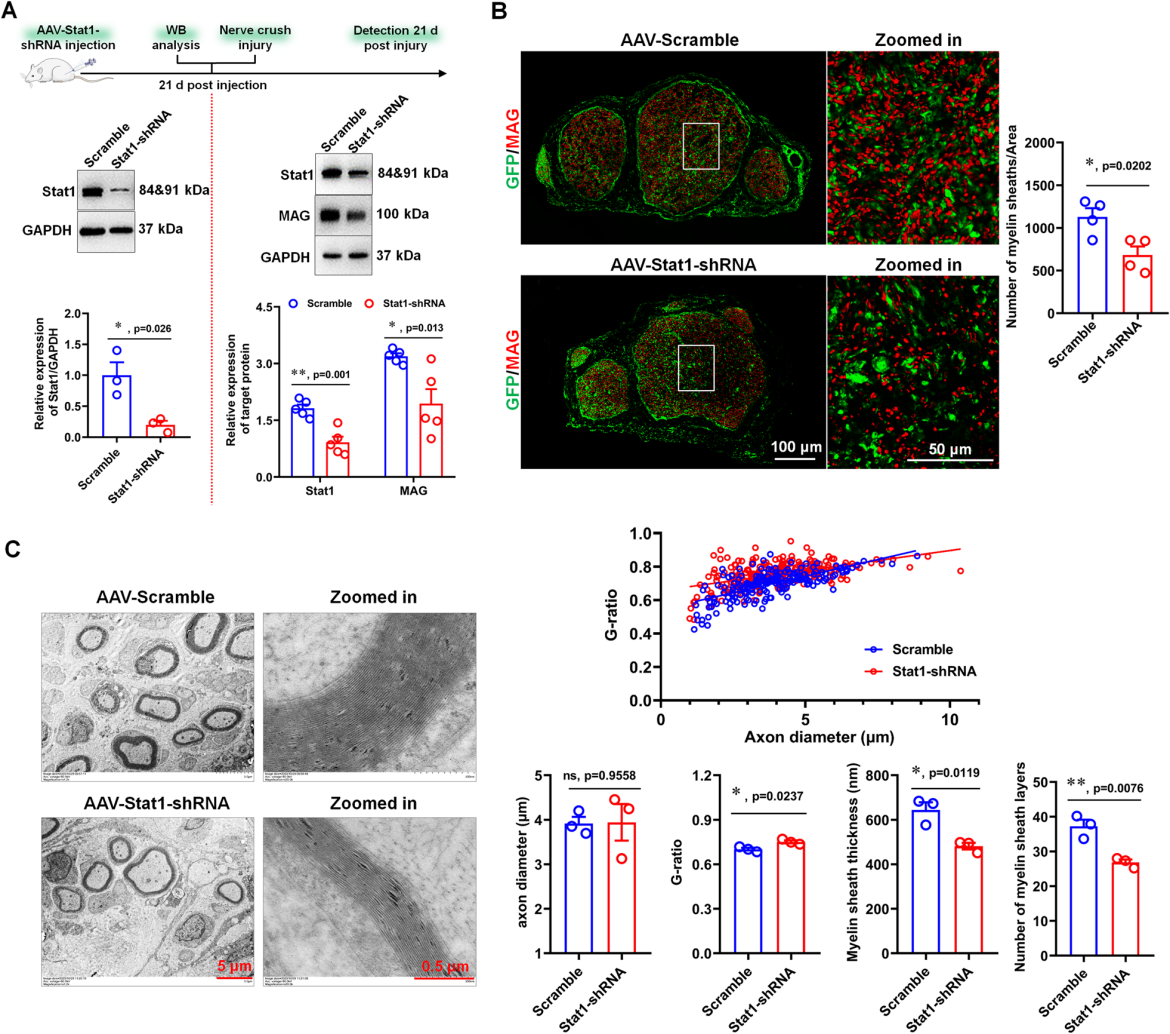
Knockdown of Stat1 in sciatic nerve inhibits remyelination. **A** Western blots showed that Stat1 expression was down-regulated by AAV-Stat1-shRNA 21 days after virus injection (on the left of the red dotted line), and MAG and Stat1 levels were down-regulated in AAV-Stat1-shRNA-treated regenerated nerves 28 days after injury relative to control (on the right of the red dotted line), with GAPDH as the internal standard. *T*-test, *p < 0.05, **p < 0.01 vs control (AAV-Scramble), n = 3 per group. **B**. MAG (red) immunostaining shows myelin sheaths in regenerated nerves treated with AAV-Stat1-shRNA and AAV-Scramble. Green is EGFP on AAV. Scale bar, 100 μm. High-magnification images of boxed regions are also shown, scale bar, 50 μm. Histograms comparing the density of myelin sheaths. *T*-test, *p < 0.05 vs AAV-Scramble, n = 3 per group. **C**. Representative transmission electron micrographs of the regenerated nerve treated with AAV-Stat1-siRNA and AAV-Scramble. Scale bars, 5 μm. Zoomed in, the high magnification image Scale bars, 0.5 μm. Histograms comparing the *G*-ratio, axon diameter, myelin sheath layers and thickness. *T*-test, *p < 0.05, **p < 0.01 vs AAV-Scramble; ns, not significant, n = 3 per group

To investigate the role of Stat1 in remyelination, an in vivo model of myelin sheath regeneration was established in the rats with Stat1-knockdown in the sciatic nerve (named Stat1-NKD rats). The Stat1-NKD rats were obtained by injection viruses of carrying Stat1-shRNA (their knockdown efficacy have been validated, Additional file 1: Fig. S2) into the sciatic nerve for 21 days, WB analysis showed that Stat1 levels in sciatic nerves of Stat1-NKD rats were significantly attenuated by Stat1-shRNA (Fig. 2A). The remyelination of Stat1-NKD rat sciatic nerve was observed by WB, IHC and TEM after 21 days injury. Myelin-associated glycoprotein (MAG), a marker of myelin formation, its expression level was decreased in Stat1-NKD rats compared to controls, as shown in the results of WB (Fig. 2A) and IHC (Fig. 2B). In addition, TEM showed that Stat1-NKD rats and controls both had clear basement membrane, high electron density and intact myelin sheath, but the thickness and layers of myelin sheath in Stat1-NKD rats were lower than those in controls, coinciding with the significant increase of *G* ratio in Stat1-NKD rats **(**Fig. 2C). Together, these data suggested that Stat1 affects remyelination in regenerated sciatic nerves.

### Expression and cellular localization of Stat1 after nerve injury

**Fig. 3:**
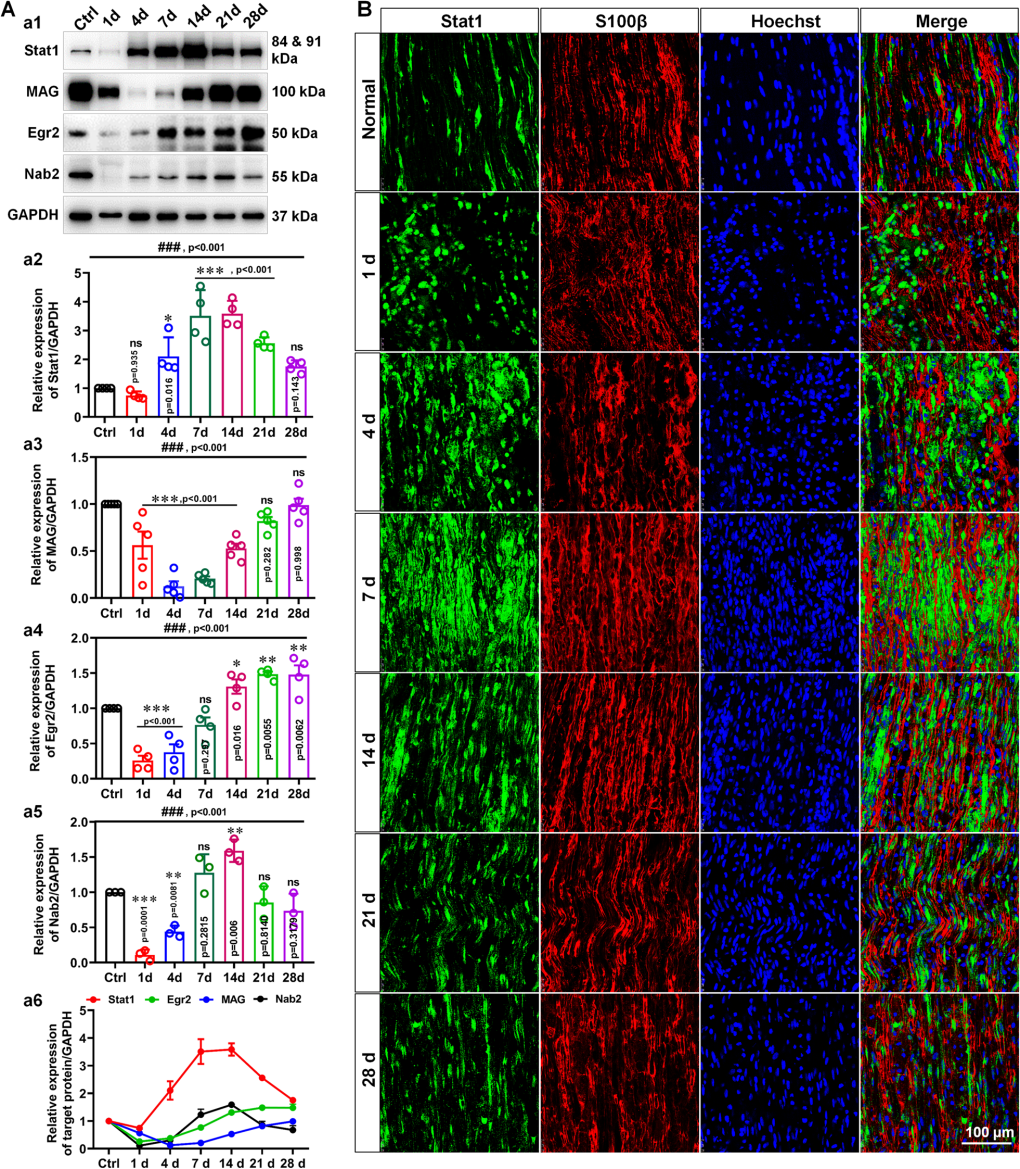
Expression changes and cellular localization of Stat1 after nerve injury.** A** (a1) Western blots showing the expression change of Stat1, MAG, Nab2 and Egr2 in nerve injury segment at the indicated time points following a nerve crush, with uninjured nerve used as the control (Ctrl). GAPDH served as the loading control. (a2–6) Histogram quantitatively compare the expression changes of Stat1, MAG, Nab2, and Egr2. ^###^*p* < 0.001, one-way ANOVA, *p < 0.05, **p < 0.01, ***p < 0.001 vs control, n = 3 ~ 4 per group. **B** Immunofluorescence staining of S100β (a marker of SCs, red) and Stat1 (green) in injured sciatic nerve showing cellular localization of Stat1: on day 1 of injury, co-localization of Stat1 and S100β was almost absent; on day 4 of injury, there was a small amount of Stat1 and S100β co-localization; and from day 7 to day 28 of injury, Stat1 was mainly localized in SCs. Scale bars, 100 μm

Given that a particular gene expressed in different cell may play different roles, we next examined the expression and cellular localization of Stat1 during regeneration after sciatic nerve injury. As shown in Fig. 3A, the expression of Stat1 reduced slightly on day 1 of nerve injury followed by a progressive increase, reached peak on day 14, and then decreased, but its level remained higher at day 28 after injury than that of normal nerves. Meanwhile, we also found that the 2 positive regulator Egr2 and Nab2, which promote and activate essential signals for SC migration, differentiation and myelination (Stolt and Wegner 2016), showed similar dynamics to Stat1, with levels decreasing on day 1 post-injury, then gradually increasing, reaching a maximum level on day 14, and then returned to normal levels. The expression of MAG showed a different pattern from those of Stat1, Egr2 and Nab2, which was down-regulated 1–4 days after injury and then increased until day 28, this is consistent with axonal demyelination early in nerve injury and subsequent axonal regeneration and remyelination.

Notably, Stat1 expressed in different cells during regeneration after nerve injury (Fig. 3B; Additional file 1: Figs. S3, S4), that is, expressed predominantly in macrophages on days 1–4 after injury (Additional file 1: Figs. S3, S4), expression in SCs from days 7 to 28 after injury (Fig. 3B; Additional file 1: Fig. S4), suggesting that Stat1 expressed in SCs may be involve in the regulation of remyelination after nerve injury, whereas Stat1 in macrophages mainly affects the inflammatory response. In addition, we observed that a significant increase of macrophages on days 1–4, coinciding with the macrophage infiltration in the early stage of nerve injury. It is well known that Stat1 regulates inflammatory responses by phosphorylating its serine at position 727 [pStat1 (Ser727)], so we examined the dynamic changes of pStat1 (Ser727) during nerve injury, and found that it increased only at day 1 (Additional file 1: Fig. S5), further confirmed that Stat1 expressed in macrophages was responsible for the inflammatory response. Together, we proposed that Stat1 may play different regulatory role during regeneration after nerve injury, that is, it influences macrophage infiltration in the early stages of nerve regeneration, and is responsible for axonal remyelination in the middle and late stages of regeneration.

### Specifically knockdown of Stat1 in SCs inhibits remyelination after nerve injury

**Fig. 4:**
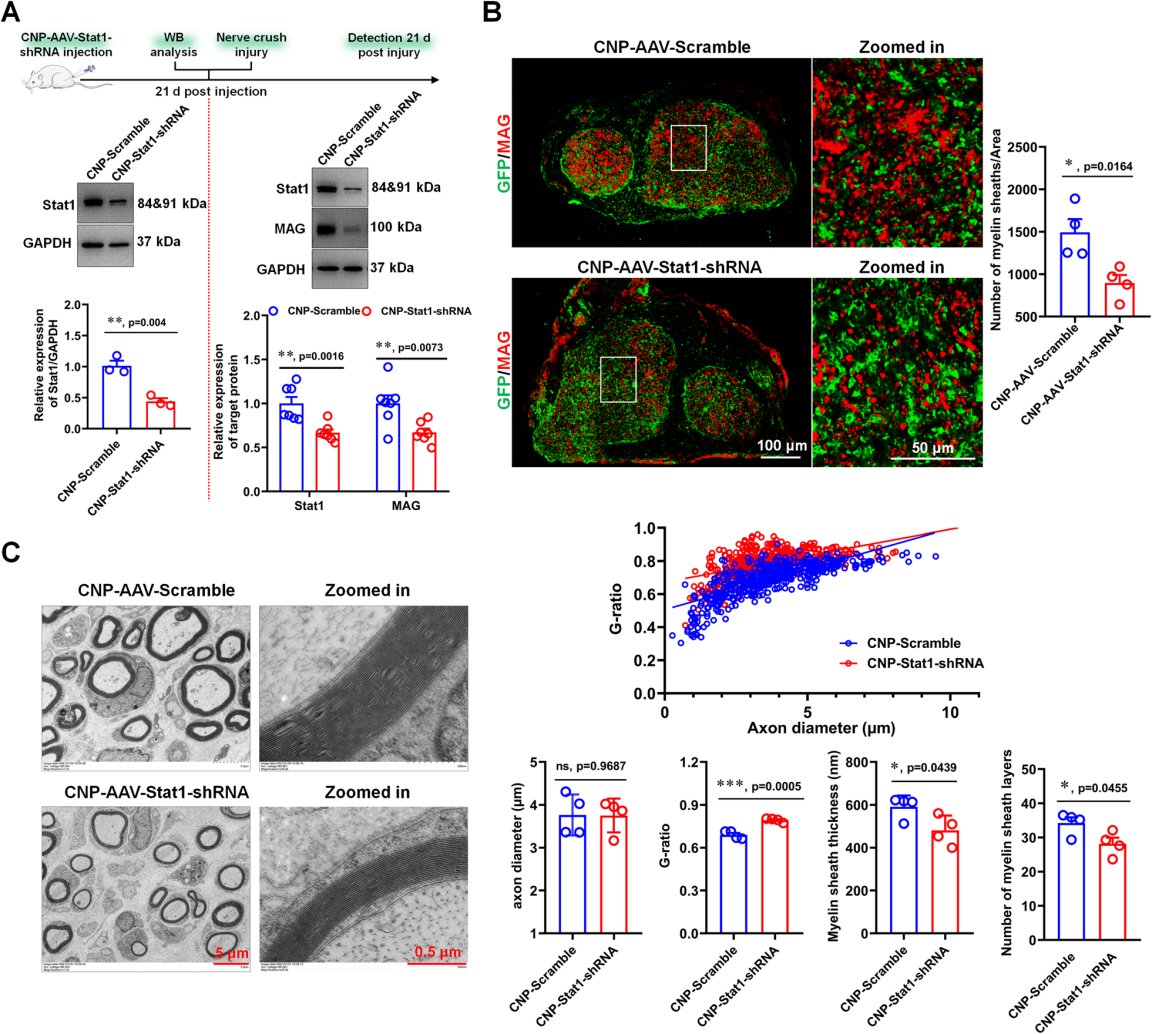
Specific knockdown Stat1 in SCs inhibits remyelination. **A** Western blots showed that Stat1 expression was down-regulated by AAV-CNP-Stat1-shRNA 21 days after virus injection (on the left of the red dotted line), and MAG and Stat1 levels were down-regulated in AAV-CNP-Stat1-shRNA-treated regenerated nerves 28 days after injury relative to control (on the right of the red dotted line), with GAPDH as the internal standard. *T*-test, *p < 0.05, **p < 0.01 vs control (AAV-CNP-Scramble), n = 3 per group. **B** MAG (red) immunostaining shows myelin sheaths in regenerated nerves treated with AAV-CNP-Stat1-shRNA and AAV-CNP-Scramble. Green is EGFP on AAV. Scale bar, 100 μm. High-magnification images of boxed regions are also shown, scale bar, 50 μm. Histograms comparing the density of myelin sheaths. *T*-test, *p < 0.05 vs AAV-Scramble, n = 3 per group. **C**. Representative transmission electron micrographs of the regenerated nerve treated with AAV-CNP-Stat1-shRNA and AAV-CNP-Scramble. Scale bars, 5 μm. Zoomed in, the high magnification image Scale bars, 0.5 μm. Histograms comparing the *G*-ratio, axon diameter, myelin sheath layers and thickness. *T-*test, *p < 0.05, **p < 0.01 vs AAV-Scramble; ns, not significant, n = 3 per group

To verify that it is indeed SC-localized Stat1 that affects myelination, we specifically knocked down Stat1 expression in SCs by injecting AAV carrying a CNPase promoter and Stat1-shRNA (CNPase-AAV-Stat1-shRNA) into the sciatic nerve. The specificity and efficacy of the CNPase-AAV transfection system were validated through examining the expression and cellular localization of green fluorescence (GFP) after 21 days of viral infection. The results showed that GFP mainly co-localized with S100β (Additional file 1: Fig. S7A), indicating that CNPase-AAV can control gene-specific expression in SCs, consistent with previous reports (Yuan et al. 2002). In addition, the Stat1 and S100β double immunostaining showed that CNPase-AAV-Stat1-shRNA drastically reduced Stat1 expression in SCs (Additional file 1: Fig. S7B), suggesting that the virus have SC specificity and efficacy. We then evaluated the effect of the SC-specific Stat1 knockdown on remyelination during nerve regeneration, and found that it was consistent with the results of Stat1 knockdown in sciatic nerve, manifested by decreased MAG levels, the number and thickness of myelin sheaths, and increased *G* ratio (Fig. 4). Collectively, these data further suggest that SC-localized Stat1 plays an important role in myelin sheath regeneration.

### Knockdown of Stat1 in SCs reduces myelination in a SC-DRG co-culture system

**Fig. 5:**
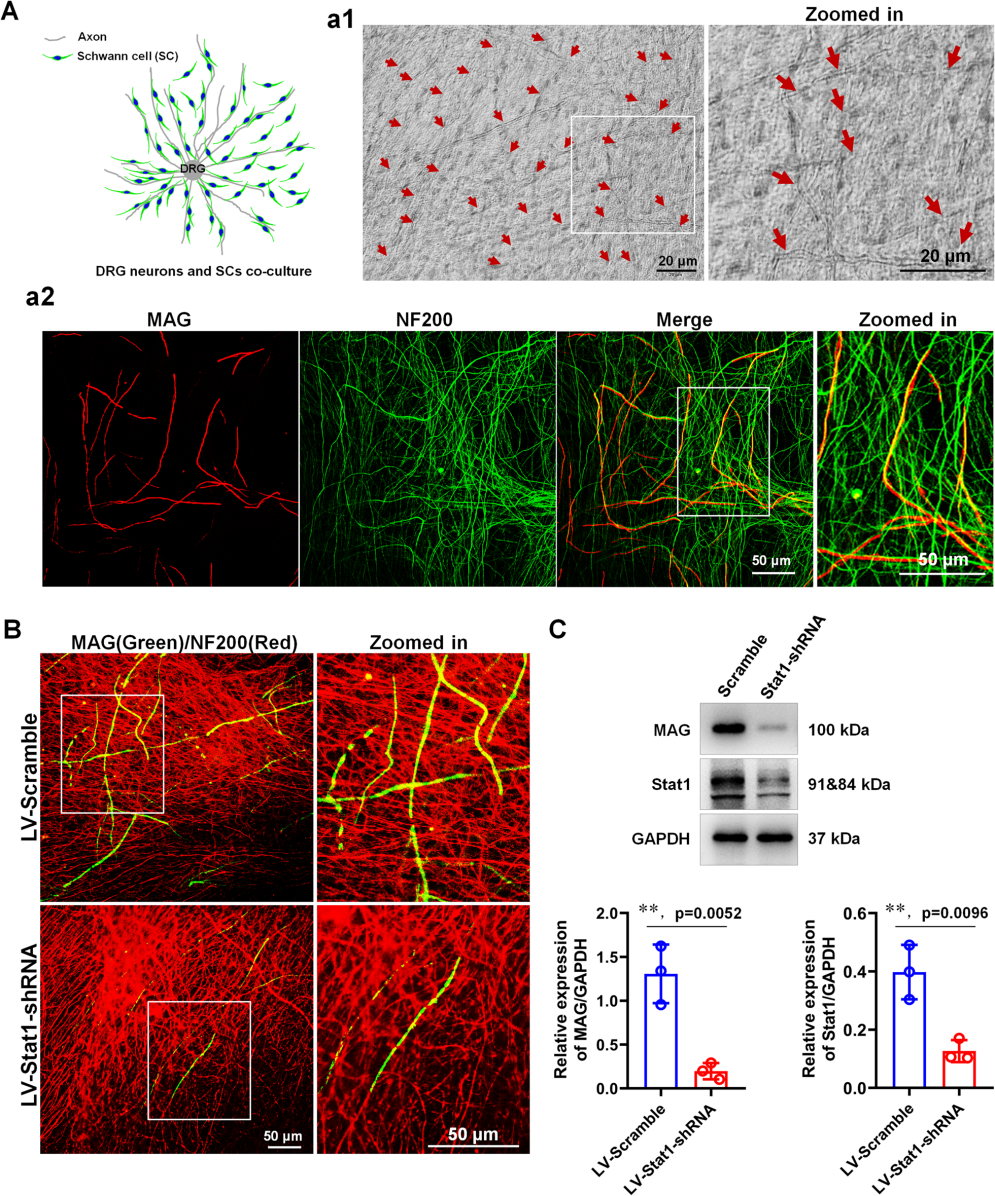
Knockdown Stat1 in SCs inhibits myelination in a DRG neurons and SCs co-culture system. **A**. Schematic showing the co-culture system; (a1) Representative phase-contrast microscopy images shows the myelin sheaths after co-culture for 21 days. Zoomed in shows high magnification image. Red arrow, myelin segments. Scale bars, 20 μm; (a2) MAG (red) and NF200 (green) immunostaining for 21 days of co-culture showed the production of numerous myelin segments. Zoomed in shows high magnification image. Scale bar, 50 μm. **B** MAG (green) and NF200 (red) immunocytochemistry indicated that knockdown Stat1 in SC with LV-Stat1-shRNA inhibits myelination co-culture systems. Zoomed in shows the high magnification images of white boxes. Scale bars, 50 μm. **C** Western blots compares the MAG and Stat1 levels in the myelin co-culture system at day 21 of Stat1 knockdown or control SCs co-culture with neurons. *T*-test, **p < 0.01 vs LV-Scramble, n = 3 per group

To further investigate the role of Stat1 in SC myelination, we co-cultured purified DRG neurons and SCs to induce myelin sheath formation, and found that most of the neuron axons were already myelinated after 21 days of co-culture (Fig. 5A), suggesting that the co-culture system could be used as a tool to study myelination in vitro. We next transfected purified SCs with a lentivirus carrying Stat1-shRNA (LV-Stat1-shRNA) or non-targeting negative control (LV-Scramble), and co-cultured them with DRG neurons 3 days after transfection to observe the effects of Stat1 on myelination. IHC and WB results showed that Stat1 knockdown inhibited the expression of MAG and the amount of myelin sheaths, indicating that SC myelination was impeded (Fig. 5B), further suggesting that Stat1 in SCs is indeed participated in the myelination.

### Knockdown of Stat1 reduces SC differentiation and promotes SC migration and proliferation

**Fig. 6:**
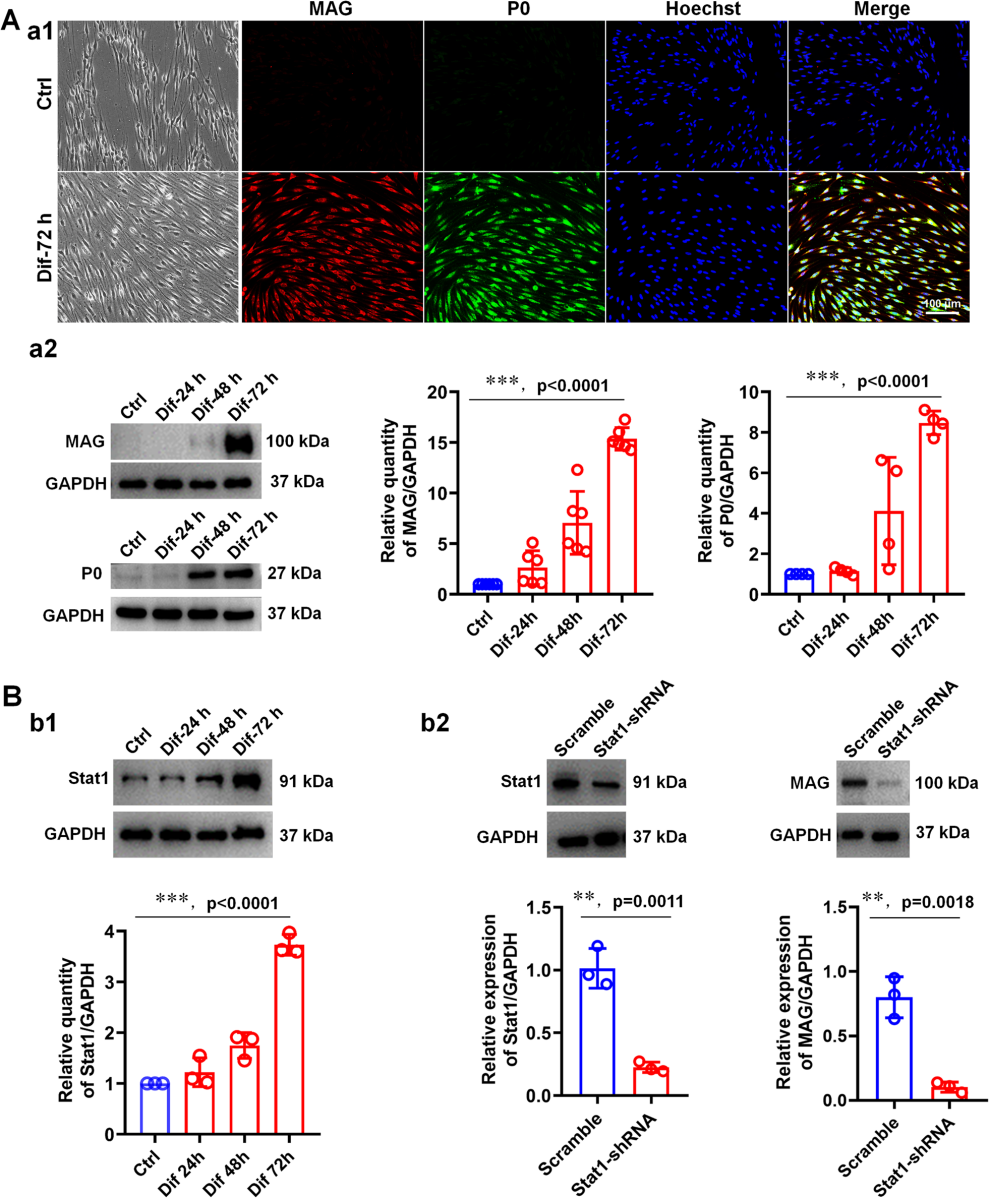
Knockdown Stat1 in SCs reduces SC differentiation. **A** (a1) Representative phase contrast microscopy images and P0 (green) and MAG (red) immunocytochemical images of SCs before and after differentiation. Scale bar, 100 μm; (a2) Western blots comparison of MAG and P0 levels in differentiated SCs at 24, 48 and 72 h, and vehicle as the control. One-way ANOVA, *** p < 0.001, n = 3 per group. **B** (b1) Western blots comparison of Stat1 levels in differentiated SCs at 24, 48 and 72 h, and vehicle as the control. One-way ANOVA, *** p < 0.001, n = 3 per group; (b2) Western blots showing the expression level of Stat1 and MAG in differentiated SCs treated with Stat1-shRNA and Scramble. The histograms showing that knockdown Stat1 reduces MAG expression in differentiated SCs. *T*-test, **p < 0.01 vs Scramble, n = 3 per group

SC differentiation is the most critical step for myelination (Simons and Trotter 2007). We used a SC differentiation assay as previously (Bacallao and Monje 2015) to explore the effect of Stat1 on SC differentiation. Following 3 days of induction, the shape of SCs changed from spindle to flattened, and the expression of myelin protein MAG and P0 increased significantly, as indicated by ICC and WB (Fig. 6Aa1–2), indicating that SCs have successfully differentiated. Furthermore, we also found that Stat1 expression increased with SC maturation (Fig. 6Bb1), suggesting that high expression of Stat1 may promote SC differentiation. Next, we observed the effect of Stat1 on SC differentiation after knockdown of Stat1 in SCs by Stat1-shRNA, and found that the differentiation capacity of Stat1-knockdown SCs was significantly reduced, manifested as its MAG expression was substantially lower than that of controls (Fig. 6Bb2), suggesting that Stat1 is involved in the regulation of SC differentiation.

**Fig. 7:**
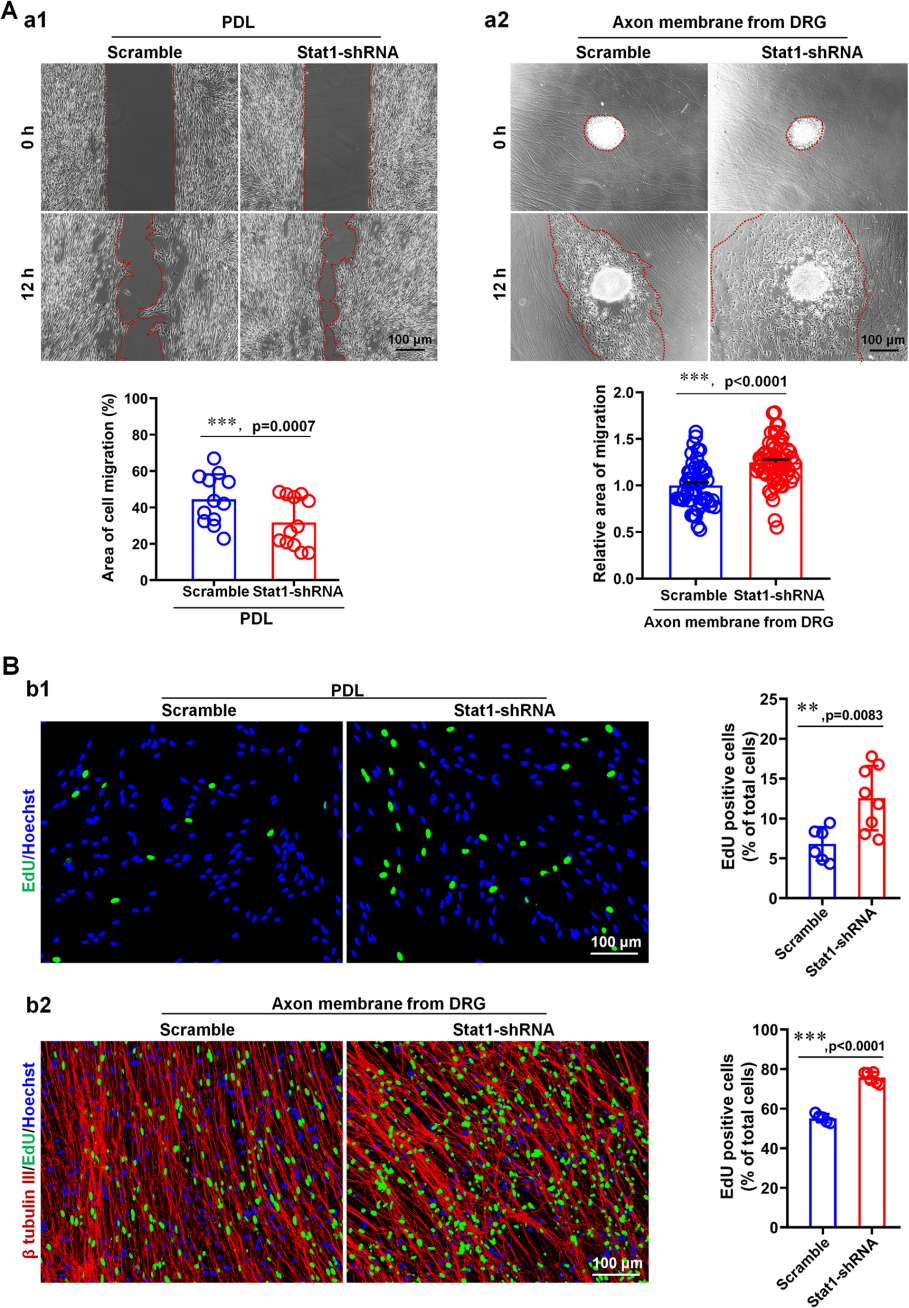
Stat1 knockdown in SCs enhances SC migration and SC proliferation. **A** Knockdown Stat1 in SCs promotes SC Differentiation. (a1) An insert-based scratch migration assay (n = 12, *t*-test, *** p < 0.001 vs Scramble) and (a2) a migration assay of cell spheroids growing on fascicular DRG axons (n = 50, *t*-test, *** p < 0.001 vs Scramble) showing SCs transfection with Stat1-siRNA have higher migratory capacity than cells transfected with a scramble. Scale bars, 100 μm. Red dashed circles indicate the extent of SC migration. Scale bars, 100 μm. **B** Knockdown Stat1 in SCs promotes SC proliferation. Results showing that SCs transfected with Stat1-siRNA growing on PDL (b1; n = 6, *t*-test, **p < 0.01 vs Scramble) or on fascicular DRG axons (b2, n = 5, *t*-test, ***p < 0.001 vs Scramble) have the higher proliferation ratio than that of scramble treated SCs. Green dots, proliferating SCs; blue dots, all cell nuclei; and red, NF200, fascicular DRG axons. Scale bar, 100 μm

Given that SC proliferation and migration are also associated with myelination (Salzer and Zalc 2016), and taking into account the inpacts of axonal signals, we cultured SCs on 2 surfaces (i.e. PDL and axon membrane) to examine the effects of Stat1 on SC migration and proliferation. For migration, we found that knockdown Stat1 in SCs could promote SC migration by using the (1) insert-based cell scratch assay (Fig. 7Aa1) and (2) cell spheroid migration assay (Fig. 7Aa2). Specifically, in the scratch assay, the wound area of SCs transfected Stat1-siRNA was approximately two-fold less than that of the scramble-siRNA treated SCs. Similar to the scratch assays, the cell spheroid migration assay showed that Stat1 knockdown enhanced the ability of SCs to migrate outward from the cell sphere regardless of being cultured on fasciculated DRG axons or PDL. These observations suggested that endogenous Stat1 in SCs may negatively regulate SC migration.

The effect of Stat1 on SC proliferation was evaluated using EdU labeling proliferation assay. Stat1-knockdown SCs and control SCs were seeded on PDL or fascicular DRG axons for 24 h before EdU labeling and cell counting. Results showed that the number of EdU and Hoechst double-labeled cells was significantly higher in Stat1-knockdown SCs than in control SCs (Fig. 7B), suggesting that endogenous Stat1 may inhibit SC proliferation, regardless of the presence or absence of DRG axonal effects.

Therefore, we speculated that Stat1 achieves regulation of myelination probably by coordinating of SC differentiation, proliferation and migration, which will be demonstrated by subsequent experiments such as RNA sequencing.

### Knockdown of Stat1 in SCs inhibits the expression of positive regulators of SC differentiation

**Fig. 8:**
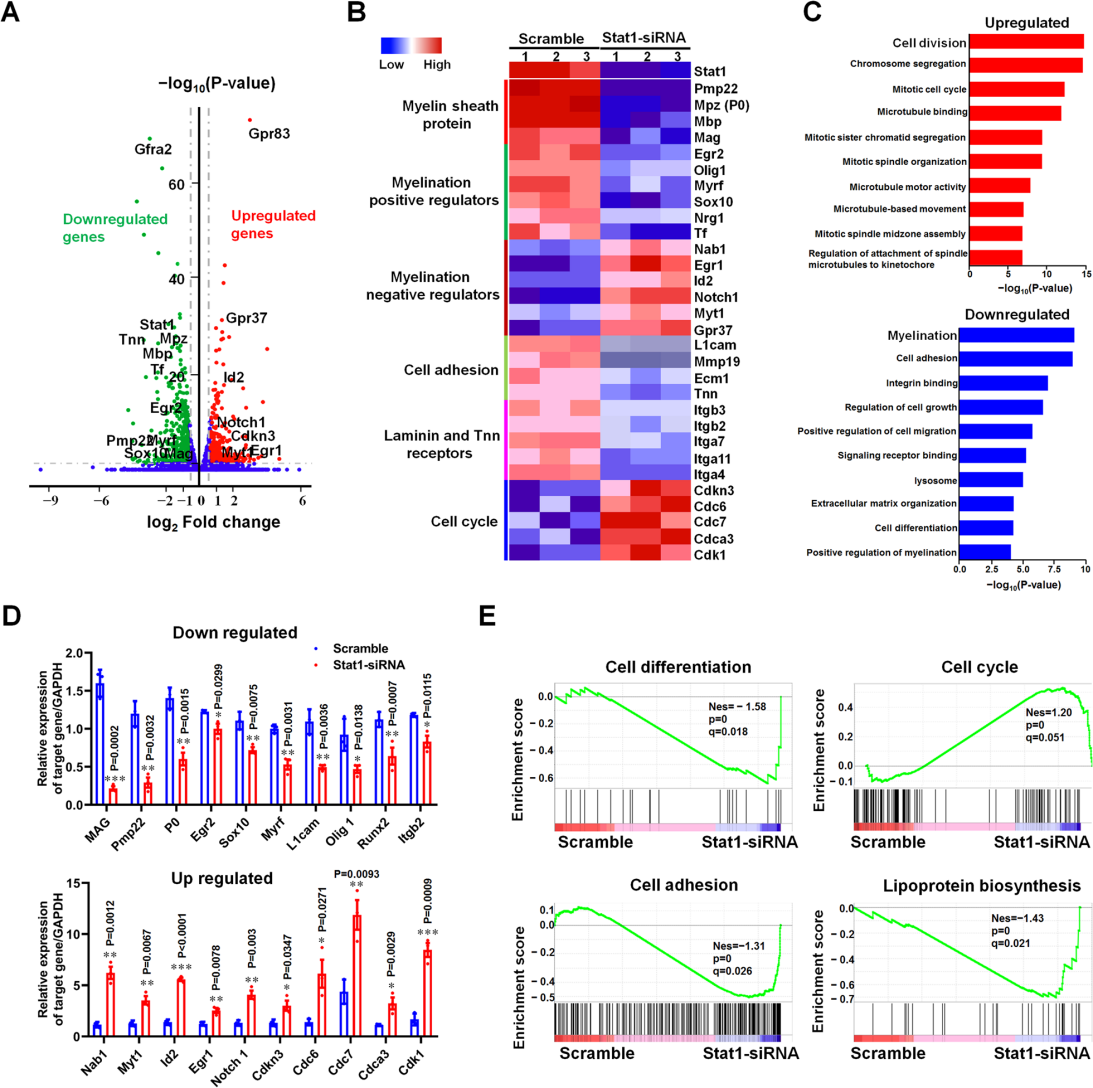
Knockdown Stat1 in SCs enhance the expression of factors negatively regulating SC differentiation.** A** Volcano plot showing the transcriptome profiles of differentiated SCs treated with Stat1-siRNA and Scramble. Red dots, upregulated genes (*p* < 0.05, fold-change > 1.5); green dots, downregulated genes (*p* < 0.05, fold-change > 1.5). **B** Heatmap of representative differentially expressed genes and their functional classification. **C** Downregulated and upregulated genes in differentiated SCs treated with Stat1-siRNA and Scramble. **D** qPCR showing that genes related to SC differentiation decreased (up) and increased (down) in differentiated SCs treated with Stat1-siRNA relative to Scramble. *T*-test, *p < 0.01, **p < 0.01, ***p < 0.01 vs Scramble, n = 3 per group. **E** GSEA enrichment scores (|NES|> 1, NOM p-value < 0.05, FDR q-value < 0.25) for cell differentiation, cell cycle, cell adhesion and lipoprotein biosynthetic process gene sets in in differentiated SCs treated with Stat1-siRNA and Scramble

To investigate the underlying mechanism by which Stat1 regulates SC differentiation, we performed RNA sequencing on 2-day-differentiated Stat1-knockdown SCs and controls, and found that knockdown of Stat1 resulted in significant changes in the expression of 1210 genes (fold change > 1.5 or < 0.66 versus control, *p-*value < 0.05; Fig. 8A; Additional file 3: Table S2.1–2.2), including downregulated positive regulators of SC myelination such as P0, Pmp22, MAG, Egr2, Sox10, Nrg1, Itgb2, and L1cam, as well as upregulated negative regulators such as Egr1, Gpr37, Notch1, Myt1, Nab1, and Id2 (Fig. 8B). GO analysis further indicated that Stat1 knockdown significantly downregulated gene functions of myelination and cell differentiation, whereas upregulation of genes related to cell proliferation (Fig. 8C; Additional file 3: Table S2.3–2.4). Results of qRT-PCR confirmed that differentiation inhibitors, such as Notch1, Id2, Myt1, Egr1 and Nab1, were highly expressed in Stat1-knockdown SCs, while positive regulators of myelination including Pmp22, MAG, Egr2, Sox10 and Runx2 were under-expressed (Fig. 8D). GSEA results also evidenced that in Stat1-knockdown SCs, genes classified as participated in myelination (i.e. cell differentiation and lipoprotein biosynthesis) were down-regulated and genes related with cell cycle were up-regulated (Fig. 8E; Additional file 3: Table S2.5).

### Stat1 may recruit Rab11fip1 to regulate the differentiation of SCs

**Fig. 9:**
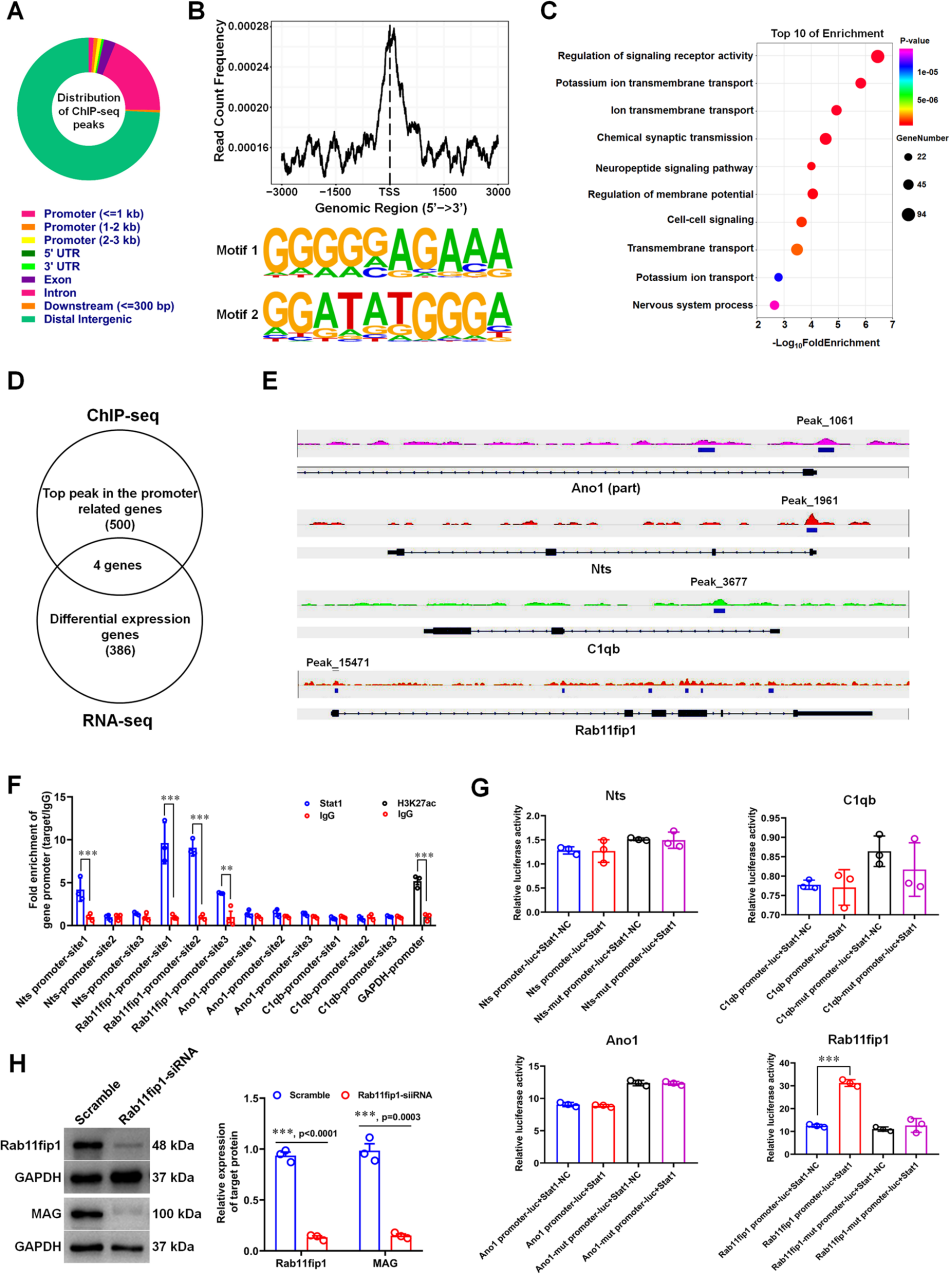
Stat1 may recruit Rab11fip1 to regulate SC differentiation. **A** Stat1-binding regions obtained by ChIP-seq. The colored rectangle annotation represent peaks at the promoter (defined as ≤ 1000 bp), the promoter (defined as 1000 − 3000 bp), downstream of thepromoter (defined as ≤ 300 bp), 5’ UTR, 3’UTR, exon, intron or distal intergenic. **B** Distribution of enrichment intensity of ChIP-seq reads in the proximity of the transcription start site (TSS) (up), and consensus motifs at Stat1 bound sequences (down). **C** GO enrichment analysis of peak-related genes (bubble diagram). **D** Venn diagram shows overlap of genes with top 500 peak in the promoter related genes and 386 differentially expressed genes (*p* < 0.05 and Fold change ≥ 2) between Stat1 knockdown groups and the control groups. **E** Histograms visualizing Stat1 binding around the 4 overlapping genes (Ano1, Nts, C1qb and Rab11fip1) locus. “Peak number” represents the plausibility ranking of the peak. **F** ChIP-qPCR for Stat1 occupancy on the Ano1, Nts, C1qb and Rab11fip1 promoters in differentiating SCs. *T*-test, **p < 0.01, ***p < 0.01, n = 3 per group. **G** Luciferase activity of Ano1, Nts, C1qb and Rab11fip1 promoter and mutant promoter in HEK293 cells co-transfected with pGV141-Stat1 (Stat1 overexpression plasmid) plus an plasmid containing the Renilla luciferase gene.* T*-test, ***p < 0.01, n = 3 per group.** H** Western blots showing the expression level of Rab11fip1 and MAG in differentiated SCs treated with Rab11fip1-siRNA and Scramble. The histograms showing that knockdown Rab11fip1 reduces MAG expression in differentiated SCs. *T*-test, ***p < 0.001 vs Scramble, n = 3 per group

We performed ChIP-Seq on 30 h-differentiated-SCs to obtain genes that are directly targeted by Stat1 in regulating SC differentiation (Fig. 9A; Additional file 4: Table S3). Stat1 was found to have an obvious signal peak within ± 3 kb near the transcription start site (Fig. 9B), indicating that Stat1 can bind to the promoter of target genes. We further executed GO analysis on 2094 target genes whose promoters can be significantly enriched by Stat1 (peak score > 40), revealing that these genes were primarily involved in crucial transmembrane transport, cell–cell signaling and chemical synaptic transmission (Fig. 9C; Additional file 4: Table S3.3). Next, we cross-referenced ChIP-seq (top 500 peak in the promoter-related genes) and RNA-seq data (386 differentially expressed genes with Fold change ≥ 2 and* p* < 0.05), four targets (Ano1, Nts, C1qb and Rab11fip1) that may be directly regulated by Stat1 were obtained (Fig. 9D–E). We then performed ChIP-qPCR and luciferase gene reporter assays to confirm the interaction of Stat1 and its target genes. Results of ChIP-qPCR showed that amplified fragments from the promoters of Nts and Rab11fip1 could be enriched by Stat1 antibody (Fig. 9F); Luciferase gene reporter assays revealed that only co-transfection Stat1 and Rab11fip1 promoter resulted in a significant upregulation of luciferase activity (Fig. 9G). These data suggested that Stat1 may regulate SC differentiation by regulating the transcriptional expression of Rab11fip1. To prove this, we acquired Rab11fip1-knockdown SCs and induce these SC differentiation and found that, similar to Stat1-knockdown SCs, Rab11fip silencing downregulated the expression of differentiation-associated gene MAG (Fig. 9H), indicating that Rab11fip does play a role in SC differentiation. Taken together, we deemed that Stat1 may recruit Rab11fip1 to regulate SC differentiation.

## Discussion

In the present study, we found a novel essential regulator involved in SC differentiation and myelination, the signal transducer and activator Stat1, by comparative analysis of transcriptomic data form myelinating SCs (Fig. 1; Additional file 2: Table S1). We found that Stat1 expression was upregulated during SC differentiation and myelin regeneration. Knockdown of Stat1 in SCs resulted in decreased remyelination after nerve injury. Loss of Stat1 in SCs prevented its differentiation and myelination, but promoted immature SC proliferation and migration, suggesting that Stat1 may play multiple roles during myelination.

To explore the effects of Stat1 on myelination, we first examined remyelination after sciatic nerve injury in which Stat1 expression was knocked down by injecting viruses carrying specific shRNAs (Fig. 2; Additional file 1: Fig. S2). We observed that Stat1-knockdown decreased the SC myelinating capacity, resulting in low MAG expression and hypomyelination (Fig. 2). Given that Stat1 is widely expressed in various cells and tissues, we examined Stat1 expression changes and cellular localization in nerves at different time points of injury to observe whether Stat1 in SCs affects myelination (Fig. 3). Stat1 was found to rarely localize in SCs (4.67 ± 0.882%) at 1 day after nerve crush, but its expression was rapidly upregulated and localized in part to SCs (31 ± 2.517%) from day 4 following injury (i.e., at the onset of remyelination), whereas Stat1 was almost entirely localized in SCs (92.3 ± 1.856%) from days 7 to 28 after injury, suggesting that Stat1 expressed in SCs plays a major role in regulating remyelination (Fig. 3; Additional file 1: Fig. S4). In addition, we also asked which cells were Stat1 mainly localized to on day 1 and 4 after nerve injury and what role did it play? The results of double immunostaining for Stat1 and F4/80 (a marker for macrophage) showed that more than 90% co-localization of Stat1 and F4/80 was observed on day 1 after nerve injury (Additional file 1: Fig. S3–S4), indicating that Stat1 was expressed by macrophages, coinciding with the macrophage infiltration in the early stage of nerve injury. It is well known that Stat1 function in inflammatory responses is largely dependent on the phosphorylation of serine 727 near its C terminus (Decker and Kovarik 2000; Levy and Darnell 2002; Shuai 2003). We therefore detected the dynamic changes in phosphorylation of Stat1 (pStat1(Ser727)) at different time points following nerve crush, found it increased only at day 1, which further confirmed that Stat1 expressed in macrophages was responsible for the inflammatory response (Additional file 1: Fig. S5). Hence, we speculated that Stat1 located in different cells is responsible for different functions after nerve injury, that is, Stat1 in macrophages regulated inflammation depending on Ser727 phosphorylation, whereas Stat1 in SCs affected remyelination in a phosphorylation-independent manner. Previous evidences suggested that macrophages interact with SCs, regulate SC migration, mitosis, and differentiation, facilitating the clearance of damaged nerve debris and subsequent regeneration, including the remyelination (Tzekova et al. 2014; Stratton et al. 2018). Therefore, here we also asked whether the macrophages can stimulate SCs high expression of Stat1 to influence the SC functions such as myelination. We treated cultured macrophages with 100 ng/ml lipopolysaccharide (LPS) for 24 h to mimick inflammatory stimulus to obtain type M1 macrophages (Additional file 1: Fig. S6A), and then co-cultured the M1 macrophages with SCs using Transwell (0.4 μm pore size, Corning), the Stat1 expression in SCs was detected by qPCR after 24 h co-culture. It was found that the level of Stat1 in SCs co-cultured with M1 macrophages was significantly higher than that in SCs cultured alone (control), suggesting that the macrophage infiltration early in nerve injury may stimulate the high expression of Stat1 in SCs (Additional file 1: Fig. S6Bb1). Furthermore, to exclude the increased Stat1 levels in SCs due to its proliferation, we detected the effect of co-culture with M1 macrophages on SC proliferation, and found that co-cultured with macrophages resulted in a significant reduction in the proliferative capacity of SCs compared with the controls, indicating that the increased Stat1 synthesis in SCs was responsible for the higher levels of Stat1 in SCs co-cultured with macrophages (Additional file 1: Fig. S6Bb2). It is well known that the pro-nerve repair state of macrophages/SCs is produced by the integration of pro-and anti-inflammatory activating signals (Wofford et al. 2021). Stratton et al. reported that macrophage-derived multiple factors including IL-6 and Gas6, play a direct role in regulating SC proliferation, survival, and remyelination (Stratton et al. 2018). Therefore, we hypothesized that the infiltrating M1 macrophages early in the nerve injury influence the SC differentiation and myelination later on, possibly by secreting some molecules, including IL6. Taken together, we concluded that Stat1 expressed in SCs plays a major role in regulating remyelination following nerve injury, and the results (Fig. 4; Additional file 1: Fig. S7) that Stat1-specific knockdown in SCs significantly reduced myelin formation in regenerated nerves further supported the above conclusion.

Using the well-designed DRG explants/SCs co-cultures systems, we found that Stat1 knockdown in SCs resulted in significant reduction of myelin sheath number and MAG expression levels (Fig. 5), indicating that Stat1 promotes myelin formation. To further explore the mechanism by which Stat1 affects myelination, we further observed its effects on SC behaviors such as proliferation, migration and differentiation during myelination process (Figs. 6 and 7). Since signals from axons are exogenous factors that precisely regulate myelination, we therefore investigated the role of Stat1 in the proliferation and migration of SCs growing on two substrates (PDL and axons) and found that Stat1 knockdown promotes proliferation and migration, with or without DRG axons, which is consistent with Stat1 dynamic during nerve injury, that is, low expression of Stat1 1 day after injury in SCs is responsible for SC proliferation and migration, the level of Stat1 gradually up-regulated after 4 days injury in SCs is beneficial to SC differentiation and later remyelination. Additionally, we observed the effect of altered Stat1 expression in neurons on remyelination of regenerated nerve. Of note, when Stat1 was knocked down in neurons by injection of AAV-Stat1-shRNA in mouse L4-L6 DRGs, it did not affect myelin regeneration in the sciatic nerves (Additional file 1: Fig. S8), suggesting the alterations of Stat1 in DRG neurons may not be involved in the regulation of remyelination. Therefore, combined the in vivo and in vitro data, we concluded that the changes in endogenous Stat1 of SCs affect myelination or remyelination by coordinating SC proliferation, migration and differentiation.

We performed RNA-seq (Fig. 8) and ChIP-seq (Fig. 9) on differentiating SCs transfected with Stat1-siRNA or scramble-siRNA to investigate the underlying mechanism by which Stat1 regulates SC myelination, and found Stat1 deficiency resulted in the upregulation of factors involved in inhibiting differentiation, such as Id2 (Jessen and Mirsky 2008), Notch1 (Jessen and Mirsky 2008), Myt1 (Lee et al. 2019; Chen et al. 2020), Nab1 (Russo et al. 1995) and Gpr37 (Yang et al. 2016), while downregulation the promyelinating factors including Egr2, Sox10, Myrf (Muth et al. 2016; Huang et al. 2018), L1cam (Wood et al. 1990), and Transferrin (Tf) (Zywitza et al. 2018; Santiago Gonzalez et al. 2019), suggesting that Stat1 acts as a two-level switch that controls SC differentiation and myelination by inactivating differentiation-negative regulators and activating pro-myelinating factors (Fig. 8). ChIP-seq data revealed that the functions of genes recruited by Stat1 binding to promoters were mainly enriched in signaling receptor activity regulation and substance such as lipoprotein and ion transmembrane transport (Fig. 9A–C). We also found that Stat1 mainly targets the distal intergenic region of myelination-related factors such as Egr2, MBP, MPZ, PMP22, Oct6, MAG, etc. (Additional file 4: Table S3.4). These results suggested that Stat1 does not directly regulate the transcription of myelin-related factors, but may enhance their transcription and expression through other means such as binding to their enhancer. Of course, we need to prove this by further experiments, such as CHIP-Seq with histone acetyltransferases p300, H3K4me1 or H3K27ac to identify enhancers or super-enhancers. By overlapping the ChIP-seq (top 500 peaks of the promoter-related genes) and RNA-seq data (386 DEGs), and combining the results of ChIP-qPCR and luciferase gene reporter assays, we eventually identified Rab11fip1 as a target gene of Stat1 (Fig. 9D–G). Rab11fip1 (a.k.a. Rab-coupling protein RCP), which binds to Rab11 to form an RCP-Rab11 complex to regulate endocytic protein sorting as well as transport of multiple proteins, and encode multiple protein transcripts associated with the plasma membrane recycling system (Peden et al. 2004; Jin and Goldenring 2006). It is well known that the key to myelination is the temporal and spatial coordination of the transport of synthetic myelin proteins (such as MPZ, PMP22, and MAG) to the plasma membrane of the myelin cells (e.g. SCs or oligodendrocytes), that is, myelin cells achieve the specific targeting and membrane fusion of the myelin-transporting vesicles by a range of signals (e.g. small GTPase Rab family) to timely control the spatial expansion of membrane and the correct formation of myelin sheath (Bruce 2004). Our previous studies found that Rab27 subfamily, including Rab27a and Rab27b, and their multiple effectors (such as Slp2-a) are involved in lysosomal exocytosis and proteolipid protein (PLP) trafficking in SCs and oligodendrocytes during myelination (Shen et al. 2016; Su et al. 2016). Therefore, we speculated that Rab11fip may be participated in myelin protein trafficking and sorting in SCs by binding to Rab family members such as Rab11, thus affecting its differentiation and formation of myelin sheaths. Here, we found that Rab11fip silencing in SCs downregulated the expression of differentiation-associated genes MAG, suggesting that Rab11fip1 is required for SC differentiation (Fig. 9H). Taken together, we believe that Stat1 initiates or regulates SC differentiation and myelination, at least in part, by binding to the promoter of its target gene Rab11fip1.

## Conclusion

Stat1 is important for myelination. Stat1 knockdown in nerve or specific knockdown in SCs reduces the axonal remyelination in a rat model of crushed sciatic nerve. Inhibition of Stat1 or its deletion blocks in SCs blocks SC differentiation thereby inhibiting the myelination program. Stat1 interacts with the promoter of Rab11fip1 to initiate SC differentiation. In summary, our findings uncover a novel function of Stat1 to regulate SC differentiation and myelination, providing a candidate molecule for clinical intervention in demyelinating diseases such as MS.

## Supplementary Information

**Additional file 1**: **Fig. S1**. QPCR analysis of 10 gene expression dynamic in myelinating SCs. **Fig. S2**. Validation of Stat1-siRNAs interference efficiency. **Fig. S3.** Stat1 expresses and localizes in macrophages post nerve injury. **Fig. S4.** Quantitative data on the co-localization of Stat1 with S100β or F4/80 after nerve injury. **Fig. S5.** Expression dynamics of phosphorylation of Stat1post nerve injury. **Fig. S6.** Effect of macrophages on Stat1 expression in SCs and SC proliferation. **Fig. S7.** The AAV-CNP- Stat1-siRNA can specifically knockdown Stat1 in SCs. **Fig. S8.** Effect of knockdown of Stat1 in DRG neurons on remyelination in sciatic nerves.

**Additional file 2**: **Table S1.** The analysis of microarray data.

**Additional file 3:** **Table S2.** The analysis of RNA-Seq data.

**Additional file 4:** **Table S3.** The analysis of ChIP-Seq data.

**Additional file 5**: **Table S4.** Primers used in qRT-PCR, ChIP- qPCR and luciferase reporter assay.

## Data Availability

The datasets that support the findings of the current study are available from the corresponding author upon reasonable request.

## Abbreviations

NDs: Neurodegenerative diseases
Stat1: Signal transducer and activator of transcription 1
MAG: Myelin associated glycoprotein
P0: Myelin protein zero
Rab11fip1: Rab11-family interacting protein 1
TuJ1: β-III tubulin
db-cAMP: Dibutyryl cyclic AMP
Ara-C: Cytosine arabinoside
PDL: Poly-d-lysine
Nrg1: Neuregulin 1
EdU: 5-Ethynyl-2′-deoxyuridine
PNS: Peripheral nervous system
SCs: Schwann cells
DRG: Dorsal root ganglion
WGCNA: Weighted gene co-expression network analysis
GO: Gene ontology
GSEA: Gene set enrichment analysis
TFs: Transcription factors
ChIP-Seq: Chromatin immunoprecipitation sequencing
RNA-Seq: RNA sequencing
ChIP-qPCR: Chromatin immunoprecipitation and quantitative PCR
IHC: Immunohistochemistry
ICC: Immunocytochemistry
WB: Western blotting
TEM: Transmission electron microscopy
