# Supplementary material for: The transcription factor Stat-1 is essential for Schwann cell differentiation, myelination and myelin sheath regeneration
Source: Mol Med. 2023 Jun 26;29:79. doi: 10.1186/s10020-023-00667-w (PMC10291779; doi:10.1186/s10020-023-00667-w)
Supplement: Supplementary file 1 — Additional file 1: Fig. S1. QPCR analysis of 10 gene expression dynamic in myelinating SCs. Fig. S2. Validation of Stat1-siRNAs interference efficiency. Fig. S3. Stat1 expresses and localizes in macrophages post nerve injury. Fig. S4. Quantitative data on the co-localization of Stat1 with S100β or F4/80 after nerve injury. Fig. S5. Expression dynamics of phosphorylation of Stat1post nerve injury. Fig. S6. Effect of macrophages on Stat1 expression in SCs and SC proliferation. Fig. S7. The AAV-CNP- Stat1-siRNA can specifically knockdown Stat1 in SCs. Fig. S8. Effect of knockdown of Stat1 in DRG neurons on remyelination in sciatic nerves. [file 10020_2023_667_MOESM1_ESM.docx]

**Additional file 1**

**Fig. S1**

**
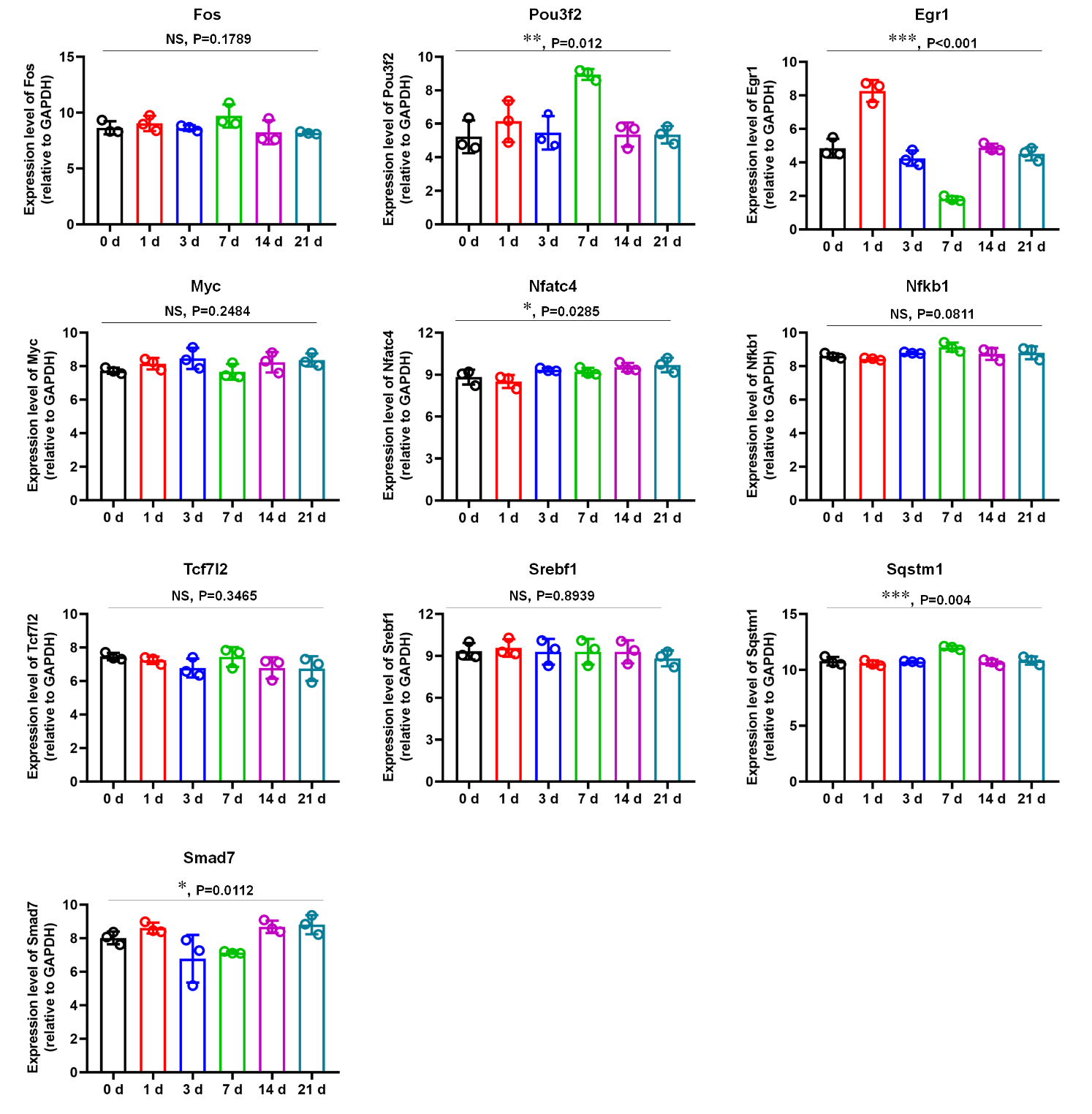
**

**Fig. S1.** **QPCR analysis of 10 gene expression dynamic in myelinating SCs**

QPCR analysis of 10 gene expression dynamic in SCs during myelination. One-way ANOVA, NS, no significantly difference; **p*<0.05, ***p*<0.01, ****p*<0.001, n=3 per group.

**Fig. S2**


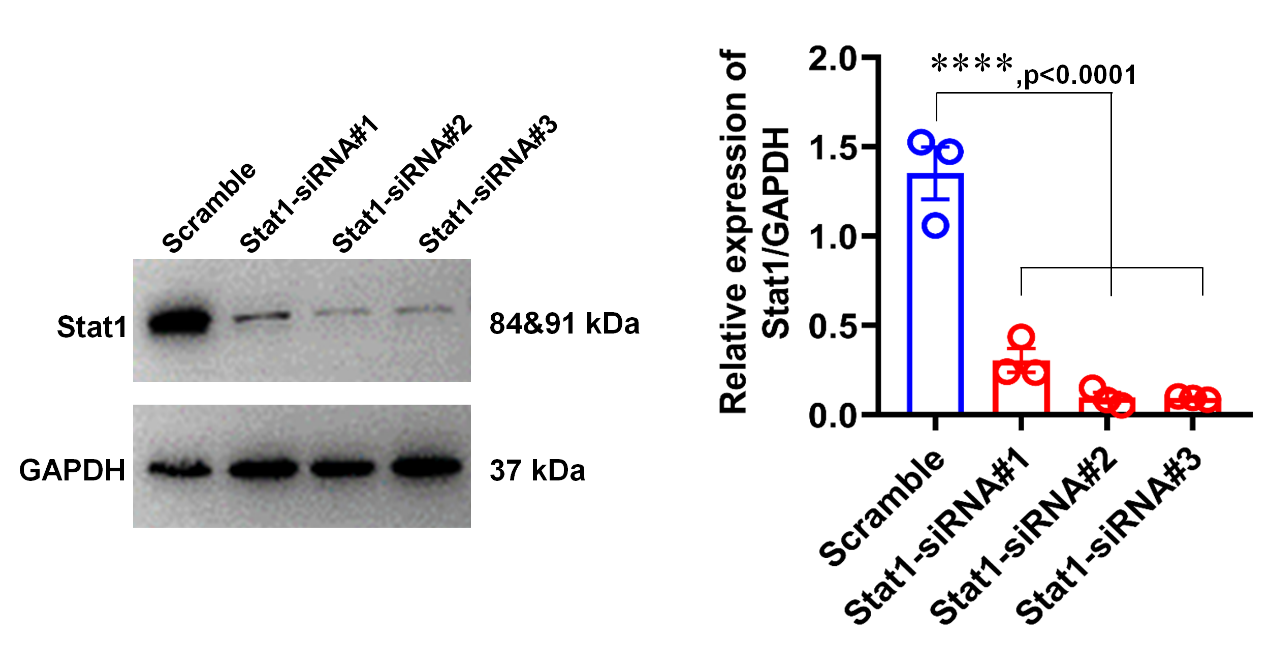


**Fig. S2.** Validation of Stat1-siRNAs interference efficiency.

Western blots comparing Stat1 levels in SCs treated with Stat1-siRNAs or Scramble for 48 hours. Histograms showing that Stat1- siRNAs significantly knockdown the expression of Stat1 in SCs. *T*-test, *****p*<0.0001 vs Scramble, n=3 per group.

**Fig. S3**


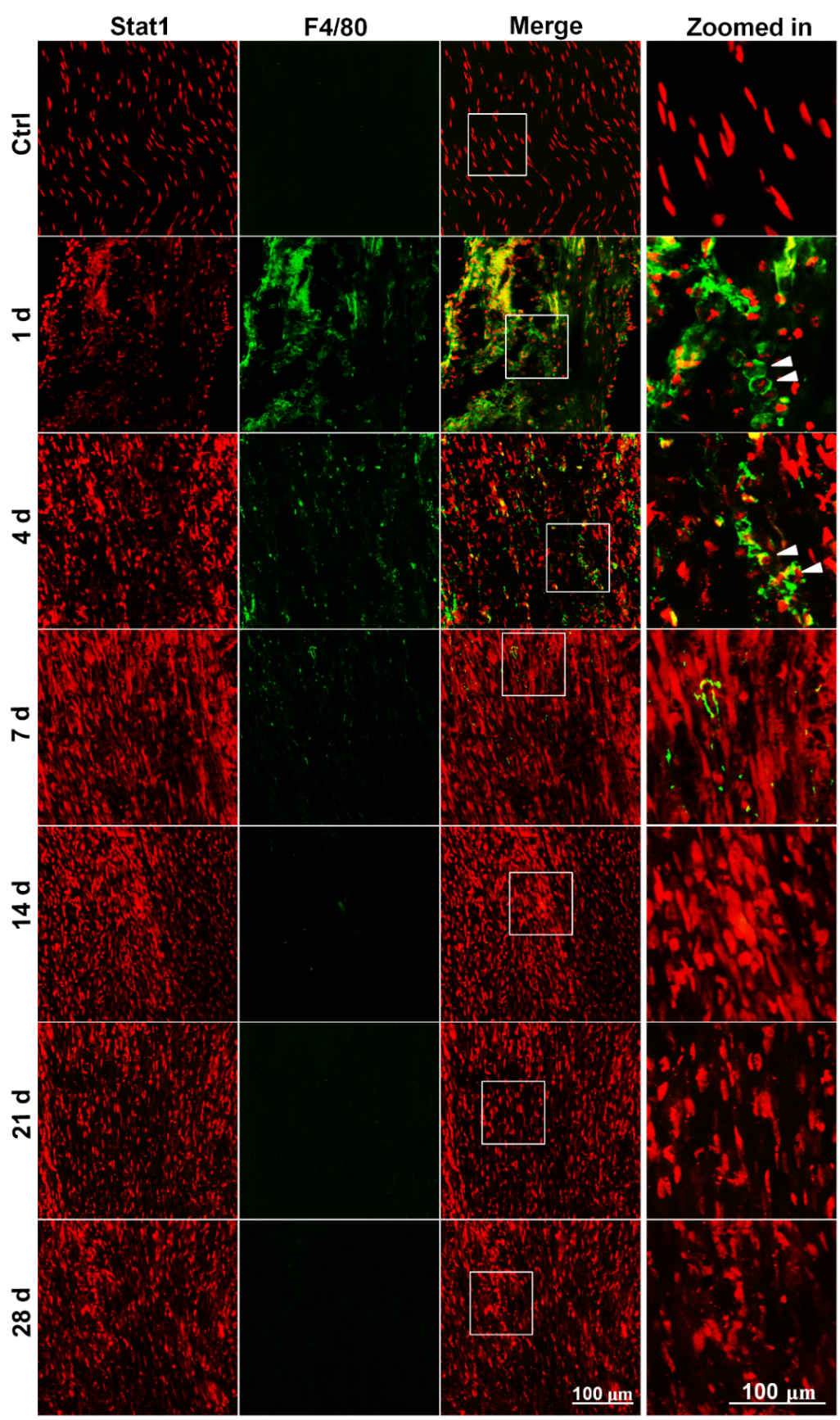


**Fig. S3. Stat1 expresses and mainly localizes in macrophages at 1-4 days post nerve injury**

Immunofluorescence staining of F4/80 (marker of macrophages, green) and Stat1 (red) in injured sciatic nerve sections. Scale bars, 100 μm.

**Fig. S4**

**
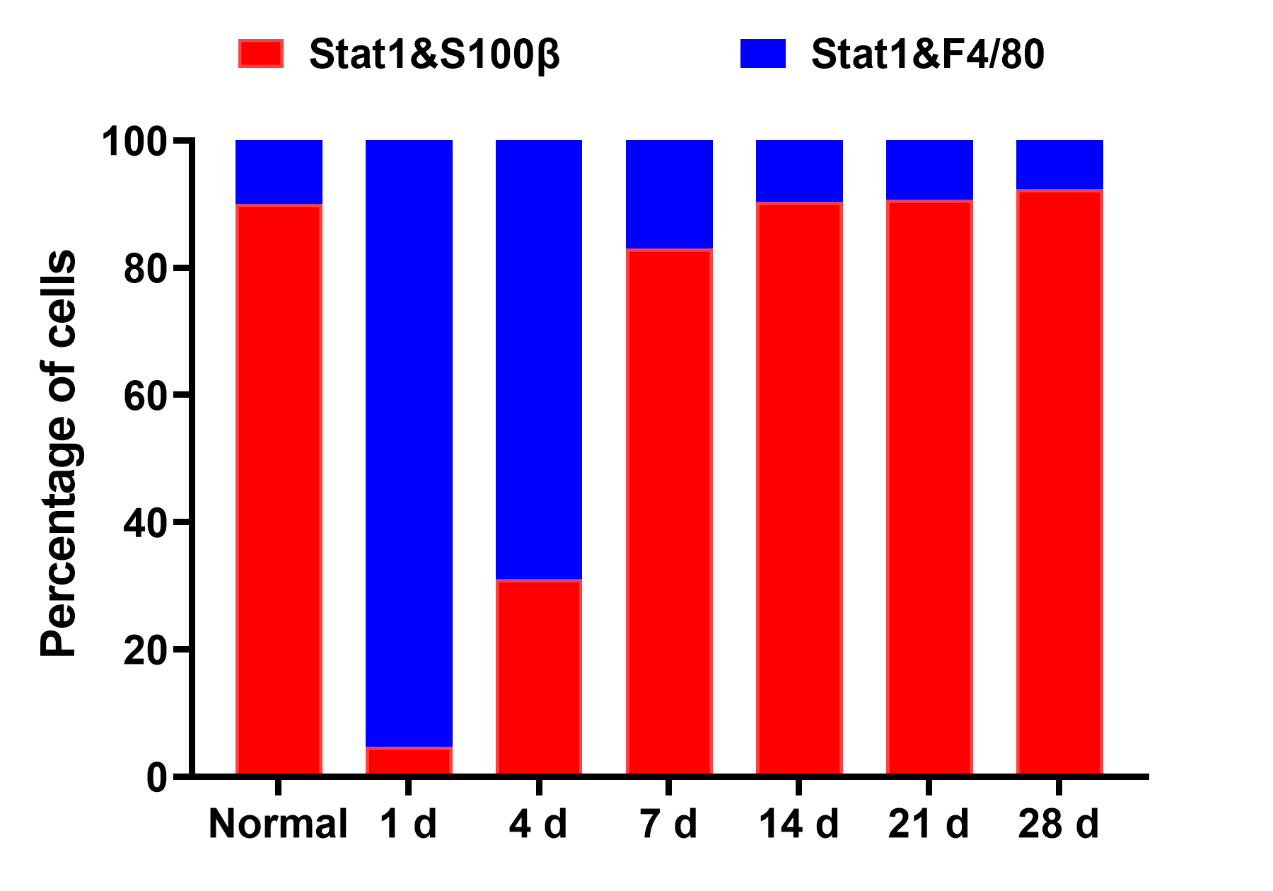
**

**Fig. S4. Quantitative data on the co-localization of Stat1 with S100β or F4/80 after nerve injury**

**Fig. S5**


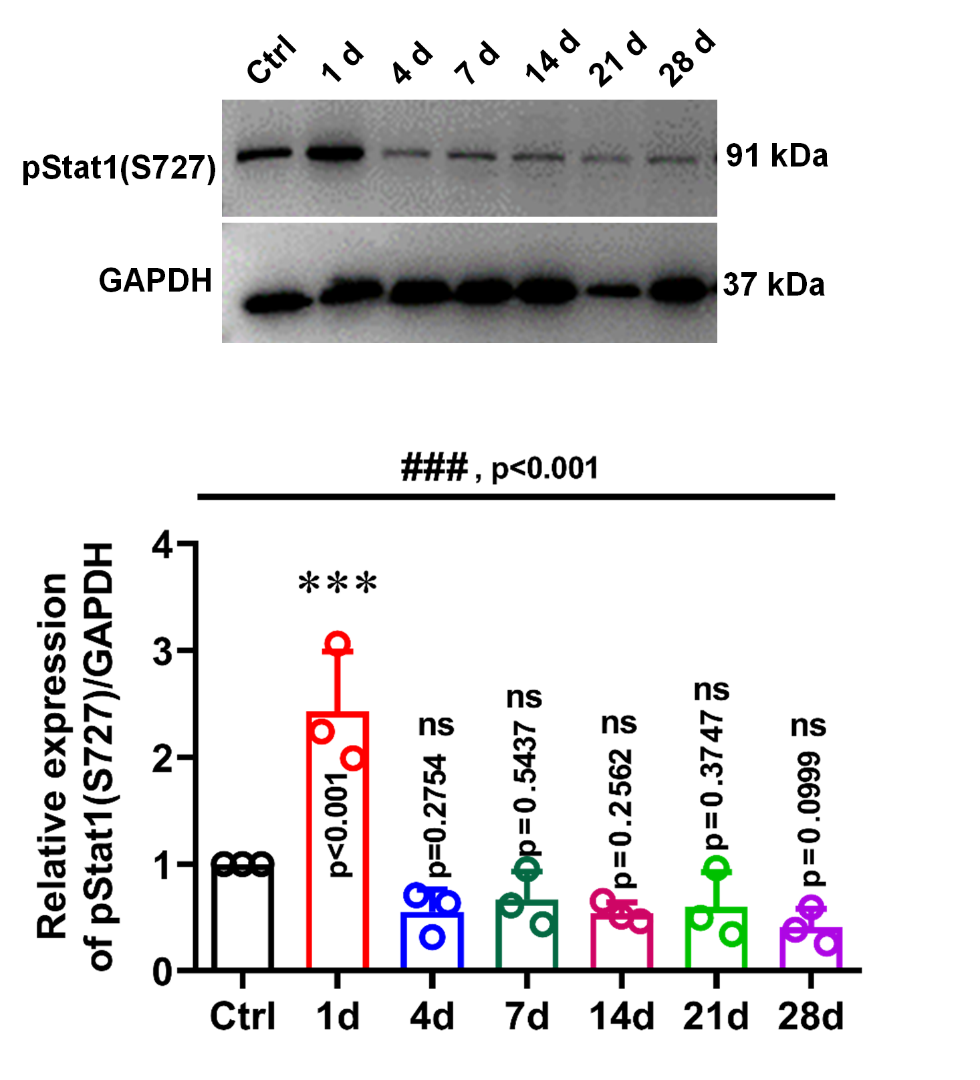


**Fig. S5.** **Expression dynamics of phosphorylation of Stat1 (pStat1 (Ser727) after nerve injury**

Western blots and histogram showing dynamic changes in phosphorylation of Stat1 (pStat1 (Ser727) in nerve injury segment at the indicated time points following a nerve crush, with uninjured nerve used as the control (Ctrl). GAPDH served as the loading control. ^###^*p* <0.001, one-way ANOVA, ****p*<0.001 vs control, ns, not significant, n=3 per group.

**Fig. S6**

**
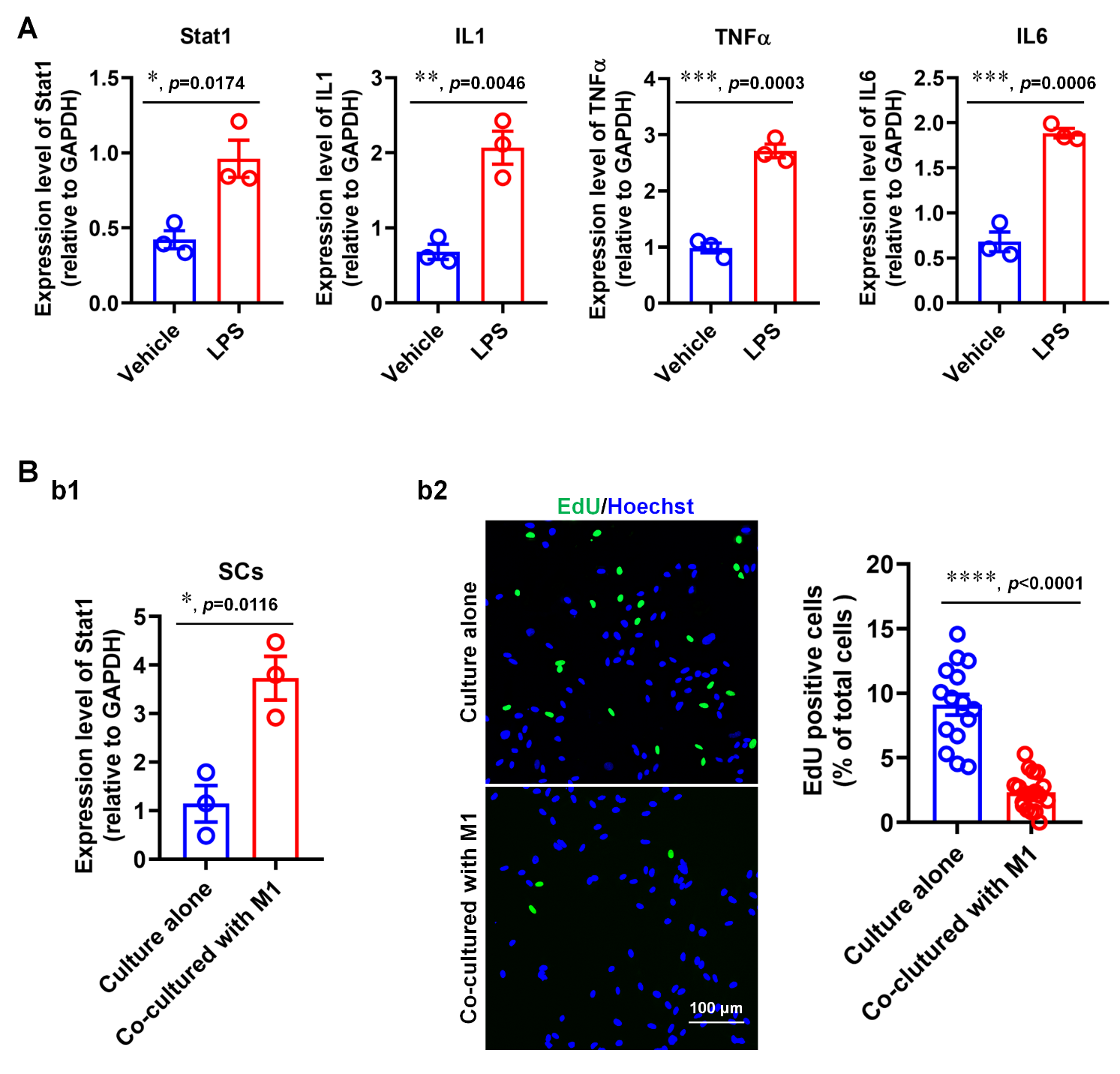
**

**Fig. S6. Effect of macrophages on Stat1 expression in SCs and SC proliferation**

**(A).** QPCR analyzed the expression of inflammatory cytokines (TNF-α, IL-6, and IL-1) as well as Stat1 in the macrophages treated with 100 ng/ml lipopolysaccharide (LPS) for 24 hours, with vehicle as control. The results showed that LPS-stimulated macrophages expressed high levels of pro-inflammatory cytokines, suggesting that the M1 type macrophages have been obtained. *T*-test, **p*<0.01, ***p*<0.05, ****p*<0.001 vs vehicle, n=3 per group. **(B).** QPCR (left) detected the expression of Stat1 in SCs co-cultured with M1 macrophages using Transwell (0.4 μm pore size, Corning) for 24 hours, and SCs cultured alone served as control. *T*-test, **p*<0.01vs control, n=3 per group. EdU labeling proliferation assay (right) was used to detect the effect of co-culture with M1 macrophages on SC proliferation, and found that co-cultured with macrophages resulted in a significant reduction in the proliferative capacity of SCs compared with the controls. Scale bar, 100 μm. Green dots, proliferating SCs; blue dots, all cell nuclei. Histograms showing the quantitative statistical data. *T*-test, *****p*<0.0001vs control, n=3 per group.

**Fig. S7**


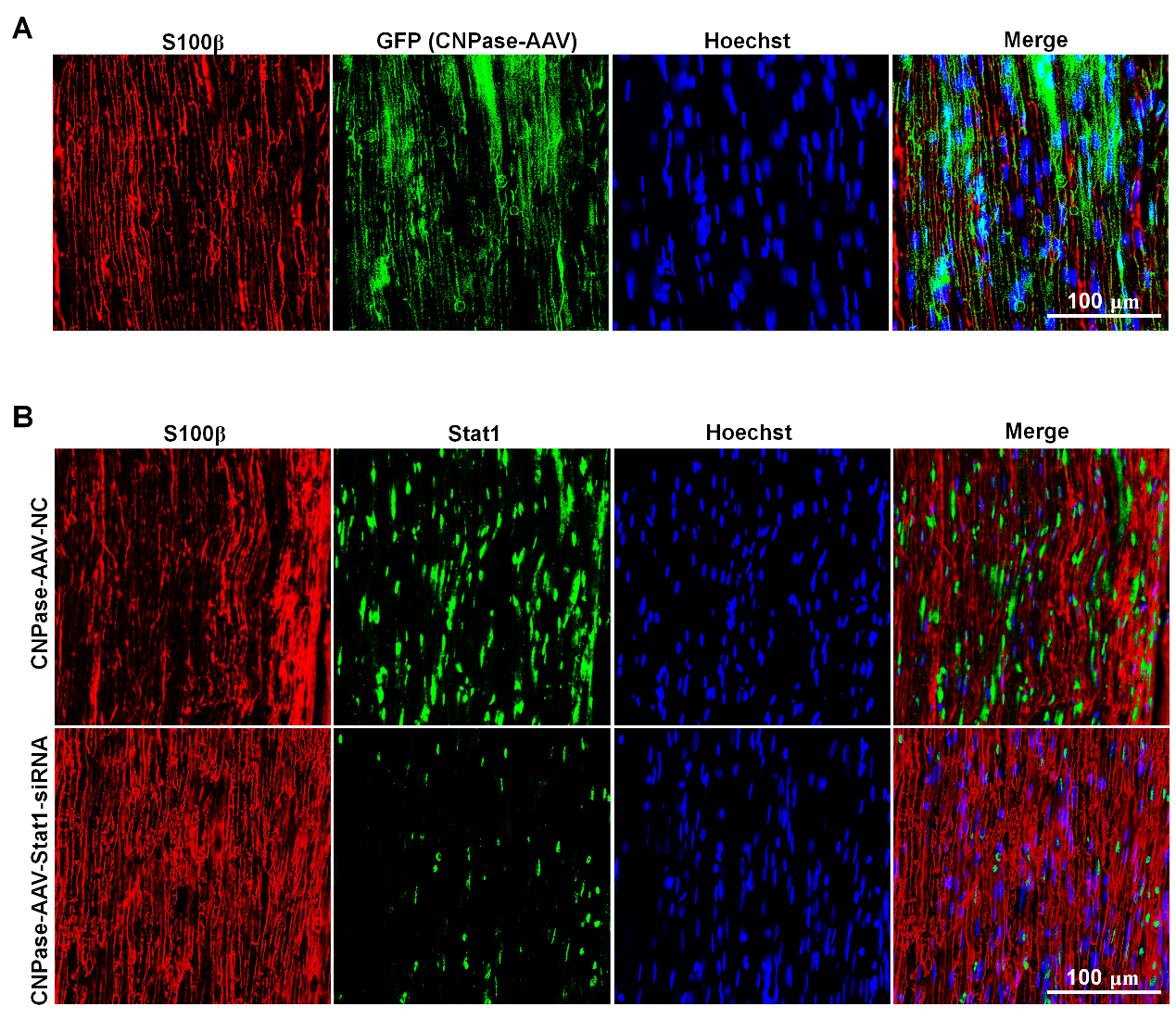


**Fig. S7. AAV-CNP-Stat1-siRNA can specifically knockdown Stat1 in SCs**

**(A).** Immunostaining with anti-S100β antibody (red), EGFP fluorescence (green) and hoechst (blue) indicating CNPase promoter can direct its downstream gene to be specifically expressed in the SCs. Scale bar, 100 μm. **(B).** Immunostaining with anti-S100β antibody (white) and Stat1 (red) indicating AAV-CNP-Stat1-siRNA actually knocks down Stat1 expression in the SCs. Blue dots (hoechst), cell nuclei. Scale bar, 100 μm.

**Fig. S8**

**
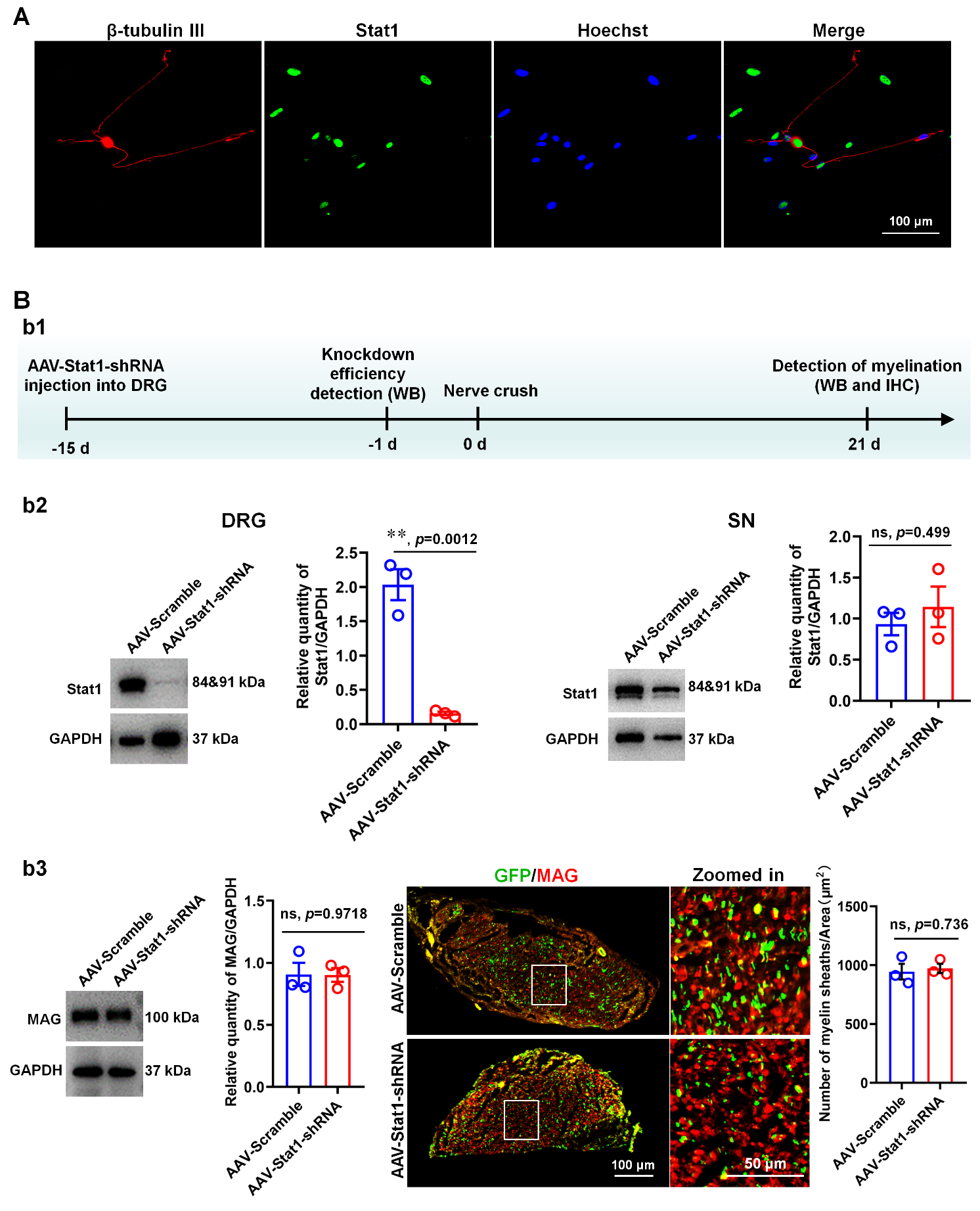
**

**Fig. S8.** **Effect of knockdown of Stat1 in DRG neurons on remyelination in sciatic nerves**

**(A).** Stat1 immunostaining on isolated cultured DRG neurons, and the results showed that expression of Stat1 was mainly localized in the nuclei of DRG neurons, whereas there was little expression in the axons. Red, β-tubulin III; Green, Stat1. Scale bar, 100 μm. **(B).** (b1) Schematic diagram illustrates the experimental process; (b2) Western blots and histogram showing the knockdown efficiency of Stat1 in DRGs (left) and sciatic nerves (SN, right) after 21 days of AAV-Stat1-shRNA injection in the DRG. *T*-test, ***p*<0.01 vs AAV-Scramble, ns, not significant, n=3 per group; (b3) Western blots (left) and immunostaining (right) with MAG antibody showing the axon remyelination in regenerated nerves treated with AAV-Stat1-siRNA and AAV-Scramble, suggesting the changes of Stat1 in DRG neurons did not affect remyelination in regenerated sciatic nerves. Scale bar, 100 μm. Also shown are high magnification images of the boxed areas, Scale bar, 50 μm. *T*-test, ns, not significant vs AAV-Scramble, n=3 per group.
