# Supplementary material for: The transcription factor Stat-1 is essential for Schwann cell differentiation, myelination and myelin sheath regeneration
Source: Mol Med. 2023 Jun 26;29:79. doi: 10.1186/s10020-023-00667-w (PMC10291779; doi:10.1186/s10020-023-00667-w)
Supplement: Supplementary file 5 — Additional file 5: Table S4. Primers used in qRT-PCR, ChIP- qPCR and luciferase reporter assay. [file 10020_2023_667_MOESM5_ESM.docx]

**Table S4. Primers used in qRT-PCR, ChIP- qPCR and luciferase reporter assay**

**Table S4.1: Sequences of siRNA used in RNA interference**

**Table S4.2: Primers used in qRT-PCR**

**Table S4.3: Primers used in ChIP-qPCR**

**Table S4.4: Primers used for expression vector construction in luciferase reporter assay**

**Table S4.1: Sequences of siRNA used in RNA interference**

| **Sequences of siRNA used in RNA interference** | |
| --- | --- |
| Name | Sequence |
| Stat1 | siRNA ^#^1: 5’- GAGGGAAATATTCAGAACA-3’ |
|  | siRNA ^#^2: 5’- GATTGGAACAGAAATTCA -3’ |
|  | siRNA #3: 5’- CCATCCTAGAACTCATTAA -3’ |
| Rab11fip1 | siRNA ^#^1: 5’- CAGGTTAATCATTATAATGAATC -3’ |
|  | siRNA ^#^2: 5’- CTCAAAAGACAAGAAGAAGAAGT -3’ |
|  | siRNA #3: 5’- GGCTACCAAAGAGACAAAAGACA -3’ |

**Table S4.2: Primers used in qRT-PCR**

| **Primers used in qRT-PCR** | | |
| --- | --- | --- |
| Name | Sequence | |
| MAG | F | 5’- TCAGGGAGACTGAGGTGAGG -3’ |
|  | R | 5’-GAAGGCTGAGATGGACGAGG -3’ |
| P0 (MPZ) | F | 5’- TCCTTCGGACACATAAGCTCC -3’ |
|  | R | 5’- GACAGAAAGATCATGTCACCGT -3’ |
| Pmp22 | F | 5’- CCATCCCTGGCTCTCGATTG -3’ |
|  | R | 5’- ACTGAAGCCATTCGCTCACA -3’ |
| Egr2 | F | 5’- AGGTTGTGCGAGGAGCAAAT -3’ |
|  | R | 5’- ATTGGGGAAGATGGTCACCG -3’ |
| Sox10 | F | 5’- ACACCTTGGGACACGGTTTT -3’ |
|  | R | 5’- GATAGGTCTTGCTCCTCGGC -3’ |
| Myrf | F | 5’- CCATGCAGGTCCAGCCAG -3’ |
|  | R | 5’- GAAGCTGGGGCTGTTTTTGG -3’ |
| Sox2 | F | 5’- AGAACTAGACTCCGGGCGAT -3’ |
|  | R | 5’- ACCCAGCAAGAACCCTTTCC -3’ |
| L1cam | F | 5’- TAGGGCCTGCAAGTGAATGG -3’ |
|  | R | 5’- TAGGGCCATAGTGCCTCCTT -3’ |
| Olig 1 | F | 5’- GTAGGCTTGTGAAGCTCGGT -3’ |
|  | R | 5’- TCGGCTACTGTCAACAACCC -3’ |
| Runx2 | F | 5’- GCTGTTGTGATGCGTATTCCC -3’ |
|  | R | 5’- GTTCTCATCATTCCCGGCCA -3’ |
| GAPDH | F | 5’- GCATCTTCTTGTGCAGTGCC -3’ |
|  | R | 5’- GATGGTGATGGGTTTCCCGT -3’ |

**Table S4.2: Primers used in qRT-PCR (Continuation table)**

| **Primers used in qRT-PCR** | | |
| --- | --- | --- |
| Name | Sequence | |
| Nab1 | F | 5’- AGCGCTCCACAAATGGTTCT -3’ |
|  | R | 5’- CAATGCTGAGTCACGAGGGA -3’ |
| Myt1 | F | 5’- ACGTGCCAAGAAGAGTGGAC -3’ |
|  | R | 5’- AGGCATATTTGCCGCTGATG -3’ |
| Id2 | F | 5’- CGGTGAGGTCCGTTAGGAAA -3’ |
|  | R | 5’- TGAGCTTGGAGTAGCAGTCG -3’ |
| Egr1 | F | 5’- AACAACCCTACGAGCACCTG -3’ |
|  | R | 5’- AAAGGGGTTCAGGCCACAAA -3’ |
| Notch1 | F | 5’- CAGTGAAGGAACGAGCCTGG -3’ |
|  | R | 5’- TTGGCTGGGAGCATCTCAAG -3’ |
| Itgb2 | F | 5’- AGGAGGAACTCCTTGGGTGA -3’ |
|  | R | 5’- TTGGTGCATTCCTCGGACAG -3’ |
| Cdkn3 | F | 5’- TGTTCTTCCCCGGATACCAC -3’ |
|  | R | 5’- CACTGCTTGTTGCCAATGCC -3’ |
| Cdc6 | F | 5’- AAGACGTCTCGCATGTCTGG -3’ |
|  | R | 5’- GGATCGGGTTTGAGGCATGA -3’ |
| Cdc7 | F | 5’- AAACTTCCGTGAACCCTGCT -3’ |
|  | R | 5’- CTCCTCCATCTGAGTCCCCA -3’ |
| Cdk1 | F | 5’- GGAACAGAGAGGGTCCGTTG -3’ |
|  | R | 5’- TGAACTGGCCAGGAGGGATA -3’ |

**Table S4.3: Primers used in ChIP-qPCR**

| **Primers used in ChIP-qPCR** | | |
| --- | --- | --- |
| Name | Sequence | |
| GAPDH promoter | F | 5’- CATGGGTGTGAACCATGAGA - 3’ |
|  | R | 5’- GTCTTCTGGGTGGCAGTGAT - 3’ |
| Rab11fip1 promoter | F1 | 5’- AGCAATCCAAGGAGTCACCG - 3’ |
|  | R1 | 5’- GAGCAGAGAGGTAAGGACGC - 3’ |
|  | F2 | 5’- GTTCCCCATCGCAGTCTCTT - 3’ |
|  | R2 | 5’- CGGTGACTCCTTGGATTGCT - 3’ |
|  | F3 | 5’- GGGGAAGCATCCTTGGAGTC - 3’ |
|  | R3 | 5’- GCGCTGCCTGAAGATGTTTT - 3’ |
| Ano1 promoter | F1 | 5’- AGGTGGTAGGACCTTCCCTG - 3’ |
|  | R1 | 5’- TTCCTCCTGGGGACAGTCAA - 3’ |
|  | F2 | 5’- GGGGACCGGAAACAATCGTA - 3’ |
|  | R2 | 5’- AGTATCCCCTGCAACCTCCA - 3’ |
|  | F3 | 5’- GCAGGGGATACTGAGAGACG - 3’ |
|  | R3 | 5’- GGTCCCTCTTGGGGGTCTAA - 3’ |
| Nts promoter | F1 | 5’- TCACCCTCCTGCTCACGTAT - 3’ |
|  | R1 | 5’- CAGCCTTCTGACAAGCAGGT - 3’ |
|  | F2 | 5’- CTGGGAGCACGATAGGGGAT - 3’ |
|  | R2 | 5’- TGTACAGGATGTGCTCTGCC - 3’ |
|  | F3 | 5’- CACGAAACACGTGCTCAGAAG - 3’ |
|  | R3 | 5’- GGCACCCCGGTACCTTTTAT - 3’ |
| C1qb promoter | F1 | 5’- TGGGATCATCTAACTGGCAAGG - 3’ |
|  | R1 | 5’- TTTCGCCAACTCAGCACCT - 3’ |
|  | F2 | 5’- ACTGGGATCATCTAACTGGCA - 3’ |
|  | R2 | 5’- ACCAGCCTGATTTCGCCAAC - 3’ |
|  | F3 | 5’- GATCATCTAACTGGCAAGGTGC - 3’ |
|  | R3 | 5’- TCCCCTCCCAAAACCCAACA - 3’ |

**Table S4.4: Primers used for expression vector construction in luciferase reporter assay**

| **Primers used for expression vector construction in luciferase reporter assay** | | |
| --- | --- | --- |
| Name | Sequence | |
| Stat1  (NM_032612) | F | 5’-TTTCTCTATCGATAGGTACCATGGAAGGAAGTGAAG  TTAAGTGAGGATATTG -3’ |
|  | R | 5’- CTTAGATCGCAGATCTCGAGGCTTAAAATCCCCTGG  ATCCCCAGCATG -3’ |
| Rab11fip1  promoter | F | 5’- TTTCTCTATCGATAGGTACCGCCCTCCCGTTCCCC  ATG -3’ |
|  | R | 5’- CTTAGATCGCAGATCTCGAGCCCAAGGTGTGCTG  CCG -3’ |
| Rab11fip1  promoter-mutant | F | 5’- TTTCTCTATCGATAGGTACCGCCCTCCCGTTCCCC  ATG -3’ |
|  | R | 5’- CTTAGATCGCAGATCTCGAGCCCAAGGTGTGCTG  CCG -3’ |
| Ano1  promoter | F | 5’- TTTCTCTATCGATAGGTACC GTCCTTAAGAGAAAA  CAAAACCAAAC -3’ |
|  | R | 5’- CTTAGATCGCAGATCTCGAG GGAACACTCTGGGG  ACCCGGGAC -3’ |
| Ano1  promoter-mutant | F | 5’- TTTCTCTATCGATAGGTACC GTCCTTAAGAGAAAA  CAAAACCAAAC -3’ |
|  | R | 5’- CTTAGATCGCAGATCTCGAG GGAACACTCTGGGG  ACCCGGGAC -3’ |
| Nts  promoter | F | 5’- TTTCTCTATCGATAGGTACC TTTTCTTTTGTCTGTT  CCCCTTCCT -3’ |
|  | R | 5’- CTTAGATCGCAGATCTCGAG CATGGGAGCCTGCT  GAAAAGC -3’ |
| Nts  promoter-mutant | F | 5’- TTTCTCTATCGATAGGTACC TTTTCTTTTGTCTGTT  CCCCTTCCT -3’ |
|  | R | 5’- CTTAGATCGCAGATCTCGAG CATGGGAGCCTGCT  GAAAAGC -3’ |
| C1qb  promoter | F | 5’- TTTCTCTATCGATAGGTACCTCTCCATGAGAGCCA  CAGAGTGTAC -3’ |
|  | R | 5’- CTTAGATCGCAGATCTCGAGCTCTACATTTTCAAA  CCCTGAG -3’ |
| C1qb  promoter-mutant | F | 5’- TTTCTCTATCGATAGGTACCTCTCCATGAGAGCCA  CAGAGTGTAC -3’ |
|  | R | 5’- CTTAGATCGCAGATCTCGAGCTCTACATTTTCAAA  CCCTGAG -3’ |
